# Lifestyle scores and their potential to estimate the risk of multiple non-communicable disease-related endpoints: a systematic review

**DOI:** 10.1186/s12889-025-21537-6

**Published:** 2025-01-23

**Authors:** Jie Ding, Ruojin Fu, Tanwei Yuan, Hermann Brenner, Michael Hoffmeister

**Affiliations:** 1 Division of Clinical Epidemiology and Aging Research, German Cancer Research Center (DKFZ), Heidelberg, Germany; 2 Medical Faculty Heidelberg, Heidelberg University, Heidelberg, Germany; 3 German Cancer Consortium (DKTK), German Cancer Research Center (DKFZ), Heidelberg, Germany

**Keywords:** Lifestyle scores, Non-communicable diseases, Cancer, Type2 diabetes, Cardiovascular diseases, Systematic review

## Abstract

**Background:**

Lifestyle scores have emerged as a practical tool to assess the risk of major non-communicable diseases (NCDs). However, most of them are primarily developed for single NCDs. Given the common risk factors for some of the major NCDs, we conducted a systematic review to evaluate the potential of existing lifestyle scores in predicting the risk of multiple NCD-related endpoints.

**Methods:**

PubMed, Web of Science, the Cochrane Library, Embase, and Google Scholar were searched from inception to October 2024. We included observational studies assessing the association between lifestyle scores and the risk of morbidity or mortality of multiple NCDs, including type 2 diabetes (T2D), cardiovascular disease (CVD), and cancer.

**Results:**

Of 16,138 unique records identified by the search, 56 eligible studies were included in the systematic review, consisting of 48 cohort studies, 5 case-control studies, 2 case-cohort studies, and 1 cross-sectional study from 16 countries. 15 lifestyle scores were identified to estimate the risk of 32 NCDs, with HLI_BMI_ being the most reported score (14/56, 25.0%). Moderate to strong associations were found between the 15 lifestyle scores and the risk of developing and dying from multiple types of cancers, CVDs, and T2D. Healthy lifestyle scores including additional risk factors (i.e., blood pressure, blood glucose, and waist circumference) aside from major risk factors (i.e., Body Mass Index (BMI), smoking, and diet) seemed to have a stronger ability to estimate NCDs risk than scores including only major risk factors.

**Conclusion:**

All 15 simple lifestyle scores were shown to estimate the risk of multiple NCDs endpoints, although some scores were originally developed to estimate the risk of single diseases only. Therefore, further research is required to identify which lifestyle score is most effective for assessing the risk of multiple NCD-related endpoints in a head-to-head comparison.

**Supplementary Information:**

The online version contains supplementary material available at 10.1186/s12889-025-21537-6.

## Introduction

Non-communicable diseases (NCDs) are the primary cause of premature death and disability in populations worldwide. In 2019, NCDs were responsible for as many as 41 million deaths (about three-quarters of all deaths), and 1.6 billion disability-adjusted life-years (DALYs) (more than 60% of the worldwide DALYs lost) [1]. In the European region, almost 90% of deaths and more than 80% of DALYs were due to NCDs [2]. Therefore, it is imperative to implement effective public policies and prevention measures to reduce the burden of these diseases.

The major NCDs, cardiovascular disease (CVD), cancer, and type 2 diabetes (T2D) are often preventable by the improvement of modifiable lifestyle behaviors, such as smoking, alcohol consumption, diet, and physical activity [3–5]. However, several of these lifestyle behaviors coexist. Therefore, only considering a single lifestyle factor is not sufficient, and a holistic assessment of multiple relevant lifestyle factors would be necessary to predict an individual’s disease risk and provide comprehensive lifestyle counseling. To this end, healthy lifestyle scores calculated from various modifiable risk factors have been developed as a cost-effective, simple, and practical tool [6–8] for single disease prediction to help identify, inform, and counsel people at high risk, and then initiate potential follow-up monitoring.

In recent years, a large number of various lifestyle scores have become available, each based on different health guidelines and calculation methods, and most of these scores were developed to predict single NCDs. However, given the common risk factors for some of the major NCDs and the simple use of lifestyle scores, it is desirable to use a single score to predict multiple NCDs. Thus, in this systematic review, we aimed to provide an overview to evaluate the potential of simple lifestyle scores to predict the risk of multiple NCD-related outcomes, including morbidity or mortality of T2D, CVD, and cancer.

## Materials and methods

This systematic review was performed according to the PRISMA guidelines [9] (the checklist can be found in the Table S1). The protocol was registered on PROSPERO (CRD42022366680).

### Search strategy

Relevant publications were identified through systematic searches of the following five electronic databases up until October 2024: PubMed, Web of Science, the Cochrane Library, Embase, and Google Scholar. We developed a search strategy with the assistance of a specialist librarian. Search terms were a combination of controlled words and free text terms on six NCDs (cancer, T2D, stroke, myocardial infarction, hypertension, and CVD), mortality, lifestyle scores, cohort study, case-control study, hazard ratio, odds ratio, and relative risk. No date, language, or other limits were set. The full search strategy is available in Material S1. After full-text screening, additional relevant studies were identified by screening the references of the studies included. Furthermore, the names of identified lifestyle scores or indexes were also searched separately to ensure a comprehensive search.

### Inclusion and exclusion criteria

Since it is unknown which lifestyle scores were used to assess various diseases, we implemented a two-step screening process. First, we included original observational studies assessing the association between lifestyle scores and the risk of morbidity or mortality for the following NCDs: stroke, myocardial infarction, hypertension, T2D, specific cancer, total cancer, and total CVD. Studies were excluded if: (1) the study did not assess one of the pre-defined endpoints; (2) the study only assessed the specific NCDs for which the lifestyle score was originally developed; (3) the lifestyle factors investigated were not combined into a lifestyle score; (4) the study did not report association estimates, including hazard ratio (HR), odds ratio (OR), and risk ratio (RR), between lifestyle scores and outcomes; (5) the study population was not adult; (6) the study subjects were patients with specific disease; (7) not peer-reviewed publications (i.e., conference abstracts, editorials, and commentaries); (8) secondary analysis. After this initial screening, we identified which lifestyle scores were used to assess the risk of multiple diseases, whether within a single study or across different studies, and excluded studies that only involved scores applied to a single disease.

### Study selection

The search results were exported into reference manager software (Endnote, version X9), and duplicate results were removed using software-supported and manual checking. After screening all titles and abstracts by two researchers independently (JD and RF), we searched the full texts of the studies retained and conducted further screening. Disagreements were resolved by discussion between the two researchers.

### Risk of bias assessment

Two researchers (JD and RF) independently assessed the quality of all included studies by using ROBINS-E tool (Risk of Bias in Non-randomized Studies – of Exposures) for observational epidemiological studies [10]. The assessment addresses bias within seven domains: (1) bias due to confounding, (2) bias arising from measurement of the exposure, (3) bias in selection of participants into the study (or into the analysis) (4) bias due to post-exposure interventions, (5) bias due to missing data (6) bias arising from measurement of the outcome, (7) bias in selection of the reported result. The risk of bias in each domain was graded as either low risk of bias, some concerns, high risk of bias, or very high risk of bias. Discrepancies were resolved by discussion or by consulting the senior investigator (MH).

### Data extraction and presentation

Two authors (JD and RF) independently extracted the following study-level data into pre-defined tables and included the following information: first author, publication year, name of the lifestyle score, population, study design, country, main results, sample size, study duration (cohort study), scoring system, the definitions of the healthy lifestyle factors, association estimates (i.e., HR, OR, RR and their 95%CIs), and characteristics of the participants (age (mean/median or range), the compositions of sex, race).

We classified the lifestyle scores and studies based on: (1) whether higher scores indicated an increasingly healthy or unhealthy lifestyle, (2) whether the lifestyle scores incorporated only five easily assessable major risk factors (smoking, alcohol consumption, diet, physical activity, and body mass index (BMI)) or whether they included the major risk factors plus additional factors or metrics (e.g., blood pressure, waist circumference, sleep).

For the presentation of the association estimates of the individual studies, we created forest plots and grouped them according to the different types of scores. We extracted HR, OR, or RR from the fully adjusted models in each study, using the highest category compared to the reference group, or per 1-point increase, to represent the relative risk between the lifestyle scores and the outcome. Due to the complexity of the different lifestyle scores’ composition and the varied outcomes assessed, we did not perform a meta-analysis. Figures were created by R software package version 4.2.1 and Adobe Illustrator 2020.

## Results

**Fig. 1:**
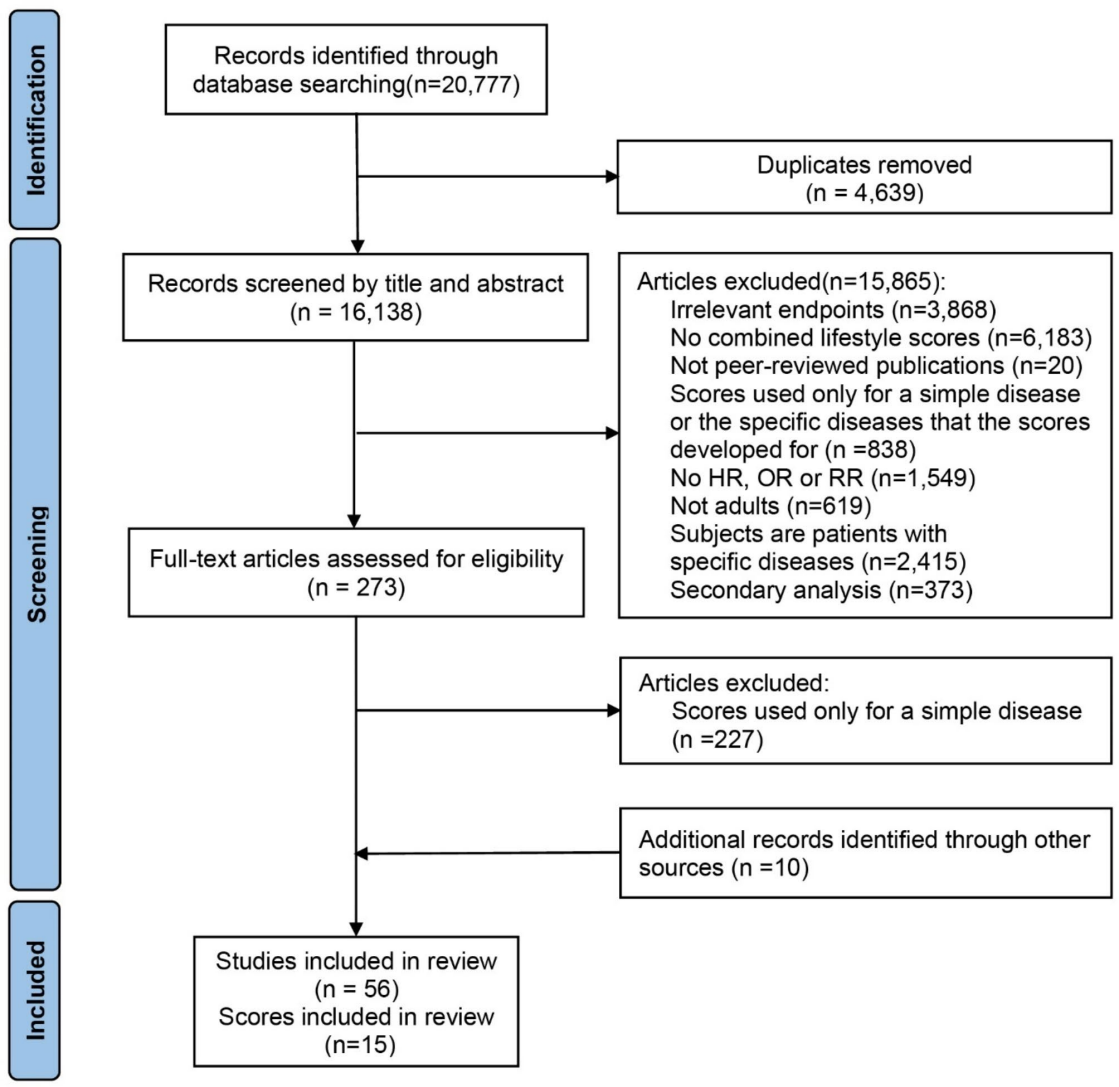
Flowchart of study selection

Our initial search identified 16,138 records after excluding duplicates (Fig. 1). After screening the title and abstract, we performed a full-text manual review of 273 articles and found 46 articles matching our inclusion criteria. In addition, 10 studies were included by searching for relevant references or names of included lifestyle scores. In the end, a total of 56 studies and 15 lifestyle scores were included in the systematic review.

### Risk of bias

The risk of bias assessment for the included studies is shown in Table S2. The overall risk of bias evaluation indicated that most of the studies showed ‘some concerns’, mainly within the measurement of the exposure and post-exposure interventions. One study was found to have a very high risk of bias because there was no description of missing values.

### Characteristics of the included studies

Table S3 summarizes the characteristics of the included articles. The 56 studies included were published from 1999 to 2024 from 16 countries. 48 (85.7%) publications were from the past 10 years, with 37 (66.1%) in 2020 or later. Of these, 5 were case-control studies (8.9%), 2 were case-cohort studies (3.6%), 1 was a cross-sectional study (1.8%), and the remaining 48 were cohort studies (85.7%). The sample size of the cohort studies varied from 1,639 to 453,808 participants, and the mean or median duration of follow-up ranged from 4.8 to 36.3 years. The included case-control studies varied in the number of cases/controls from 89/178 to 485/3763. Studies were mostly conducted in the United States (23/56, 41.1%), followed by the United Kingdom (10/56, 17.9%), and Iran (9/56, 16.1%). A total of 15 lifestyle scores were summarized in this review. Healthy lifestyle index with BMI (HLI_BMI_) was the most frequently reported score for the risk of multiple NCD-related endpoints (14/56, 25.0%). The outcomes of the studies comprised 32 types of NCDs, of which the most investigated was CVD mortality (14/56, 25.0%), followed by cancer mortality (*n* = 12/56, 21.4%), breast cancer incidence (11/56, 19.6%), and colorectal cancer incidence (11/56,19.6%).

### Components of lifestyle scores

The components of the 15 lifestyle scores are shown in Table 1, with additional details given in Table S4-Table S6. Since all the lifestyle scores including additional factors are all healthy lifestyle scores (HLS), the lifestyle scores were classified into three types of scores: HLS including major factors, unhealthy lifestyle scores (UHLS) including major factors, and HLS including additional factors. Four HLS including major factors were identified: HLI_BMI_ [11–24], the American Cancer Society guidelines score (ACS guidelines score) [25, 26], low-risk lifestyle score [27], and World Cancer Research Fund and the American Institute for Cancer Research score (WCRF/AICR score) [28, 29]. The UHLS including major factors comprised five scores: empirical lifestyle pattern score for hyperinsulinemia (ELIH) [30–37], empirical lifestyle pattern score for insulin resistance (ELIR) [31, 32, 34, 35, 38], lifestyle inflammation score (LIS) [39–48], chronic disease risk index (CDRI) [49], and the health behaviors score [50, 51], Another six HLS including additional factors were identified: life’s simple 7 (LS7) [52–56], ideal cardiovascular health metrics (ICVHMs) [57, 58], life’s essential 8 (LE8) [59, 60], healthy lifestyle index with waist circumference (HLI_WST_) [21, 22], healthy lifestyle index with waist-to-hip ratio (HLI_WHR_) [17], and the Mediterranean lifestyle (MEDLIFE) [61–65].

Among all the scores, only HLI_BMI_ included all five major risk factors, while the others included only three or four factors. LS7, LE8, and ICVHMs included some physiological metrics, such as blood pressure, total cholesterol, and fasting plasma glucose. MEDLIFE included additional lifestyle factors, such as hours of sleep, watching TV, and socializing with friends. For HLI_WST_ and HLI_WHT_, waist circumference (WST) and waist-to-hip ratio (WHR) were used to replace BMI to assess body fatness, respectively.

**Table 1:** Components of the included lifestyle scores

Lifestyle scores	Components of lifestyle scores					
	Smoking	Alcohol consumption	Diet	Physical activity	BMI	Additional factors or metrics
**HLS including major factors**						
HLI_BMI_	X	X	X	X	X	
ACS guidelines score		X	X	X	X	
Low-risk lifestyle score	X	X	X	X		
WCRF/AICR score		X	X	X	X	
**UHLS including major factors**						
ELIH		X	X	X	X	
ELIR		X	X	X	X	
LIS	X	X		X	X	
CDRI	X	X	X		X	
Health behaviors score	X	X	X	X		
**HLS including additional factors**						
LS7	X		X	X	X	Blood pressure, total cholesterol, fasting plasma glucose
LE8	X		X	X	X	Blood pressure, non-high density lipoprotein cholesterol, HbA1c, sleep
ICVHMs	X		X	X	X	Blood pressure, total cholesterol
HLI_WHR_	X	X	X	X		Waist-to-hip ratio
HLI_WST_	X	X	X	X		Waist circumference
MEDLIFE		X	X	X		Nap, hours of sleep, watching TV, socializing with friends, collective sports

HLS, healthy lifestyle score; UHLS, unhealthy lifestyle score; BMI, body mass index; ACS guidelines score, the American Cancer Society guidelines score; WCRF/AICR score, World Cancer Research Fund and the American Institute for Cancer Research score. ELIH, empirical lifestyle pattern score for hyperinsulinemia; ELIR, empirical lifestyle pattern score for insulin resistance; LIS, lifestyle inflammation score; CDRI, chronic disease risk index; LS7, life’s simple 7; LE8, life’s essential 8; ICVHMs, ideal cardiovascular health metrics; WST, waist circumference; WHR, waist-to-hip ratio; MEDLIFE, the Mediterranean lifestyle

### Associations between lifestyle scores and NCDs

**Fig. 2:**
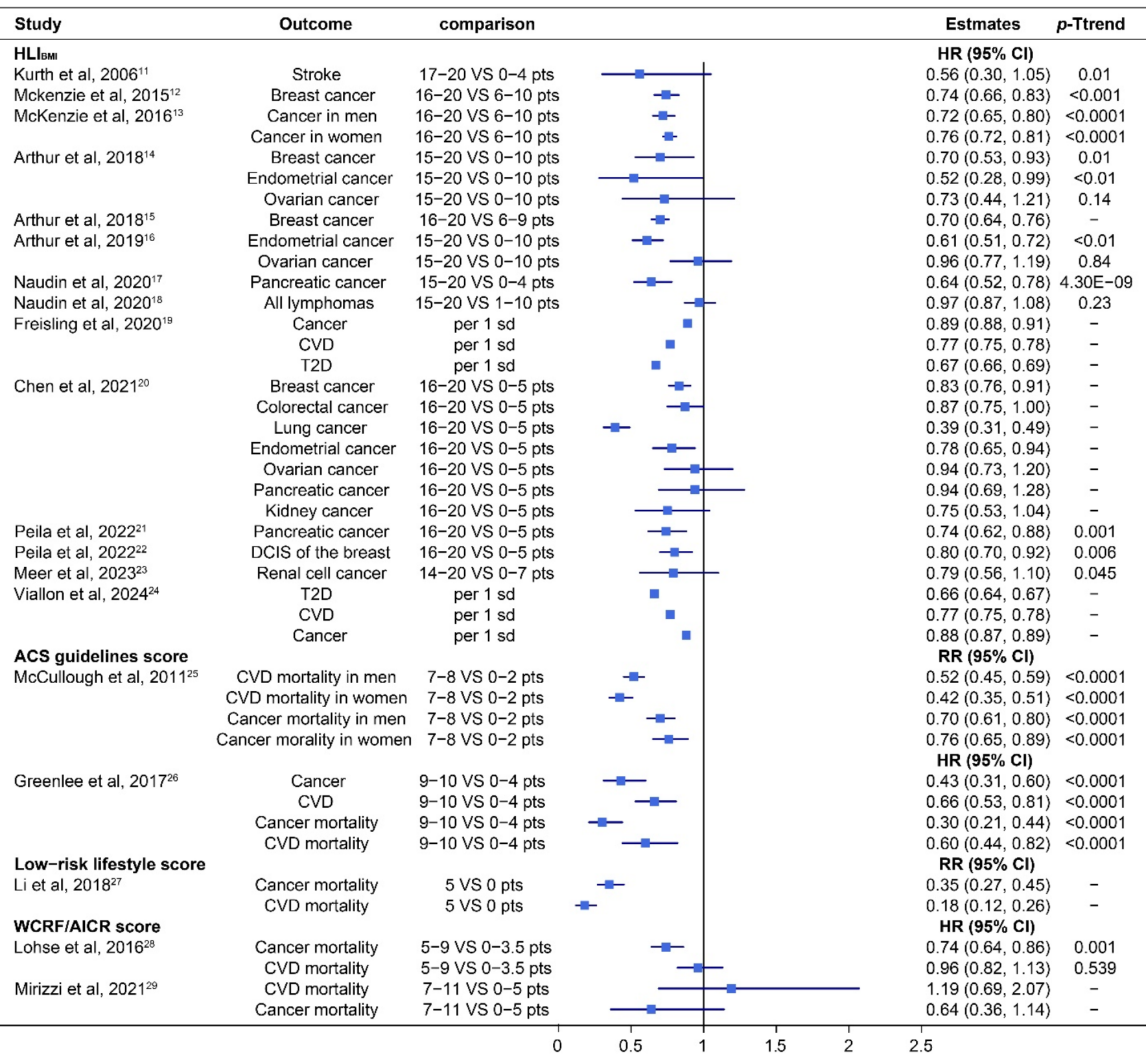
Association between HLS including major factors and the risk of multiple NCD-related endpoints

Figure 2 shows the estimates reported by 19 studies between HLS including major factors and the risk of multiple NCD-related endpoints. HLI_BMI_ showed a statistically significant association with the incidence of breast cancer (*n* = 4), endometrial cancer (*n* = 3), lung cancer (*n* = 1), with a range of 17–61% lower risks for individuals in the highest versus the lowest HLI_BMI_ quantile. There were 33–34%, 23%, and 11–12% reductions in risk of T2D, CVD, and cancer, respectively, per unit increase in the HLI_BMI_. In terms of pancreatic cancer, two of three studies [17, 21] showed a lower risk with higher HLI_BMI_. Higher scores on the ACS guidelines (*n* = 2) and low-risk lifestyle scores (*n* = 1) were associated with a lower incidence and mortality of CVD and cancer (HRs from 0.30 to 0.66, RRs from 0.18 to 0.76), while higher scores on the WCRF/AICR score (*n* = 1) were associated with lower cancer mortality (HR 0.74 95% CI 0.64 to 0.86), but it did not show a statistically significant association with CVD mortality. Most of the studies calculated *a P*-trend for scores across different categories, with 20 out of 24 (83.3%) being statistically significant.

HLI_BMI_, healthy lifestyle index with body mass index; ACS guidelines score, the American Cancer Society guidelines score; WCRF/AICR score, World Cancer Research Fund and the American Institute for Cancer Research score; CVD, cardiovascular disease; T2D, type 2 diabetes; HR, hazard ratio; RR, risk ratio; CI, confidence interval; pts, points.

**Fig. 3:**
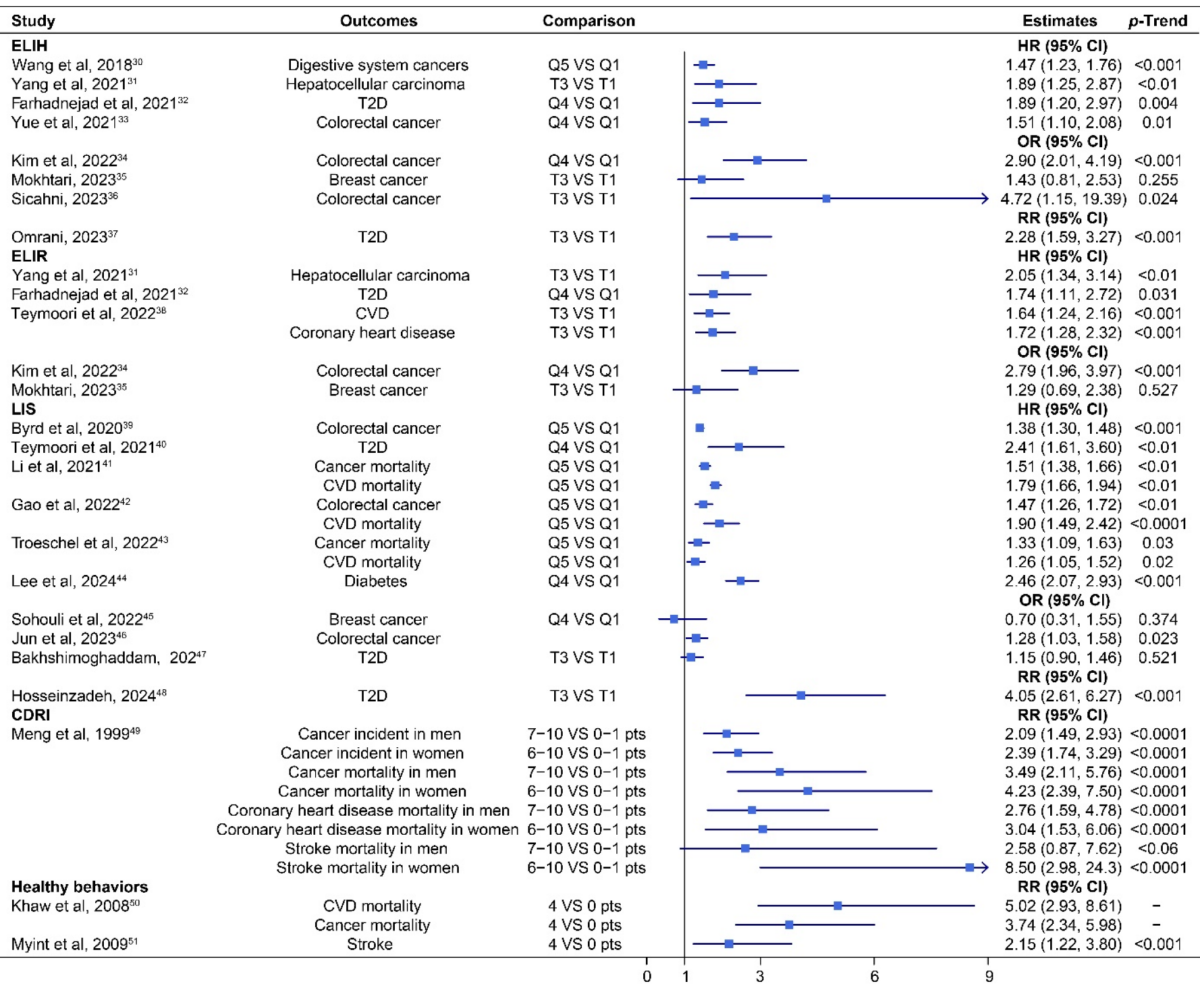
Association between UHLS including major factors and the risk of multiple NCD-related endpoints

ELIH, empirical lifestyle pattern score for hyperinsulinemia; ELIR, empirical lifestyle pattern score for insulin resistance; CDRI, chronic disease risk index; LIS, lifestyle inflammation score; CVD, cardiovascular disease; T2D, type 2 diabetes; HR, hazard ratio; OR, odds ratio; RR, risk ratio; CI, confidence interval; pts, points.

Figure 3 shows that 22 studies investigated the associations between UHLS including major factors and the risk of developing multiple NCDs endpoints. Compared to individuals in the lowest category, those in the highest category for ELIH, ELIR, LIS, CDRI, and the healthy behaviors score had a higher risk of developing total digestive system cancer (*n* = 1), hepatocellular carcinoma (*n* = 2), diabetes (*n* = 6), colorectal cancer (*n* = 7), coronary heart disease (*n* = 1), stroke (*n* = 1), cancer (*n* = 1), and CVD (*n* = 1). LIS, CDRI, and health behaviors score were associated with dying from cancer (*n* = 4), coronary heart disease (*n* = 1), and CVD (*n* = 4). For stroke mortality, the association with CDRI was statistically significant for women, but not for men. Among all the UHLS, the magnitude of the association was moderate to strong, with relative risks ranging from 1.26 to 8.50, but no statistically significant association with breast cancer risk was observed with ELIH, ELIR, and LIS, respectively [35, 45]. The risk was more than twofold in individuals in the highest categories relative to those in the lowest for half of the estimates on the incidence of colorectal cancer [34, 36], and four of seven estimates on diabetes [37, 40, 44, 48], hepatocellular carcinoma [31], cancer [66], stroke [67], as well as the mortality of coronary heart disease [66], stroke [66], cancer [66, 68], and CVD [68]. Furthermore, for all of these NCD-related endpoints, with the exception of breast cancer and stroke mortality in men, there was a statistically significant linear trend (*P*-trend < 0.05) between UHLS including major factors across the categories.

**Fig. 4:**
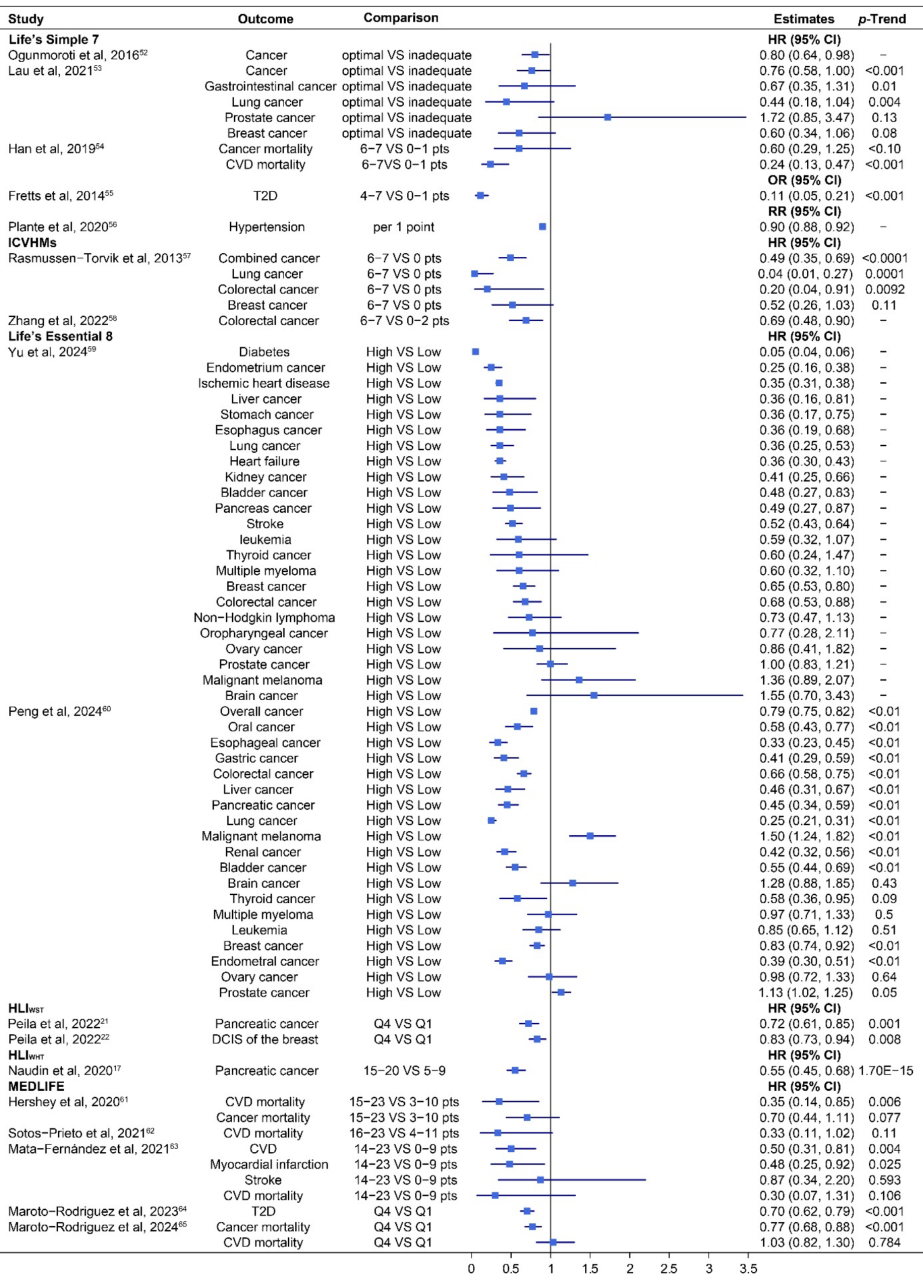
Association between HLS including additional factors and the risk of multiple NCD-related endpoints

LS7, life’s simple 7; ICVHMs, ideal cardiovascular health metrics; LE8, life’s essential 8; MEDLIFE, the Mediterranean lifestyle; HLI_WST_, healthy lifestyle index with waist circumference; HLI_WHR_, healthy lifestyle index with waist-to-hip ratio; DCIS, ductal carcinoma in situ; CVD, cardiovascular disease; T2D, type 2 diabetes; HR, hazard ratio; OR, odds ratio; RR, risk ratio; CI, confidence interval; pts, points.

Figure 4 shows the association between the identified HLS including additional factors and the risk of multiple NCD-related endpoints, which were investigated in 17 studies. Similar to Fig. 1, higher scores were significantly associated with a moderate or strong inverse relationship to multiple NCD-related risks, with point estimates ranging from 0.04 to 0.90 in 43 out of 70 results (61.4%), and a significant *P*-trend (< 0.05) in 30 out of 44 cases (68.2%). Higher scores on the LS7 were associated with a strongly decreased risk of developing cancer (*n* = 1), T2D (*n* = 1), hypertension (*n* = 1), and dying from CVD (*n* = 1) (relative risks from 0.11 to 0.90). For ICVHMs, there was an inverse association between ICVHMs and the risk of developing combined cancer (*n* = 1), lung cancer (*n* = 1), and colorectal cancer (*n* = 2) (HRs from 0.04 to 0.69). MEDLIFE was strongly associated with the risk of incident CVD (*n* = 1), myocardial infarction(*n* = 1), and T2D (*n* = 1) (HRs from 0.48 to 0.70), as well as CVD mortality(*n* = 1) and cancer mortality(*n* = 1) (HRs from 0.35 to 0.77). Both HLI_WST_ and HLI_WHR_ were significantly associated with a lower risk of developing pancreatic cancer, but the association for HLI_WHR_ (HR 0.55, 95% CI 0.45 to 0.68, *P*-trend = 1.70E-75) was stronger compared with that for HLI_WST_ (0.72, 0.61 to 0.85, 0.001).

Overall, moderate or strong associations were found between all three kinds of lifestyle scores and the risk of developing various types of cancers, including breast cancer, colorectal cancer, endometrial cancer, ovarian cancer, pancreatic cancer, lung cancer, digestive system cancer, gastrointestinal cancer, DCIS of breast, hepatocellular carcinoma, and various CVDs, including stroke, myocardial infarction, as well as hypertension and T2D. Additionally, these lifestyle scores have been associated with a decreased (HLS) or an increased (UHLS) mortality from total cancer and different CVDs, including coronary heart disease, stroke, and total CVD. Moreover, the associations between WHR-based HLI, WST-based HLI, and pancreatic cancer risk (HR 0.72, 95% CI 0.61 to 0.85; 0.55, 0.45 to 0.68) were both slightly stronger than the BMI-based HLI in the same study [17, 21] (HR 0.74, 95%CI 0.62 to 0.88; 0.64, 0.52 to 0.78) (see Figs. 2 and 4). Conversely, the association was marginally weakened when the HLI was constructed with waist circumference (HR 0.83, 95% CI 0.73 to 0.94) instead of BMI for DCIS of the breast [22] (0.80, 0.70 to 0.92, Figs. 2 and 4). Overall, the associations between HLS including additional factors with the risk of multiple NCD-related endpoints seemed to be stronger than for HLS including only major risk factors. Specifically, the risk of 28 out of 70 (40.0%) assessed NCD-related endpoints was reduced by over 50%, compared to only 6 out of 42 (14.3%) when only major factors were considered (see Figs. 2 and 4).

## Discussion

Our review identified that 15 lifestyle scores were generally associated with a reduced risk of developing and dying from various NCDs, such as cancer, T2D, and CVD. Moreover, compared with HLS including only major lifestyle factors, HLS including additional factors seemed to have a stronger ability to predict NCDs risk. These findings highlight the potential of using a simple lifestyle score in identifying individuals at high risk for multiple NCDs simultaneously in primary care or through self-testing by using online platforms.

### Principle findings and possible interpretations

Our study findings are consistent with the recent research indicating that the effect of lifestyle on NCDs is not limited to a single factor but is instead dependent on a combination of factors. Therefore, although simultaneous interventions targeting multiple lifestyles are challenging, they might be a particularly efficient approach to prevent NCDs. For instance, a systematic review by Zhang et al. [69] found that adopting multiple healthy lifestyles, measured by the WCRF/AICR score, was associated with substantial risk reduction in the risk of developing or dying from cancer. Meanwhile, a number of randomized controlled trials have yielded similar findings. A 23-year follow-up of a cluster randomized trial in Da Qing, China, showed that a six-year diet, exercise, and weight intervention resulted in lower incidences of CVD, CVD mortality, and T2D in the intervention group compared to the control group [70, 71]. However, there are no randomized controlled trials examining the impact of combined lifestyle interventions on cancer. Consequently, the summary of the relationship between lifestyle scores and cancer risk in our systematic review provides strong evidence for this gap. Furthermore, the coexistence of multiple unhealthy lifestyles has been demonstrated to have multiplicative or synergistic negative effects on health. A prospective case-control study conducted by Marrero at the University of Michigan showed a synergistic effect of alcohol, tobacco, and obesity on the risk of hepatocellular carcinoma [72]. Similarly, a case-control study from Northern Italy and Switzerland suggests that when tobacco and alcohol are taken together, their combined effect is rather multiplicative than additive on laryngeal cancer risk [73].

While few lifestyle scores were originally designed to evaluate the risk of multiple NCDs, in our review, we observed that numerous studies have made attempts to apply lifestyle scores originally developed for a particular endpoint to the assessment of the risk for other NCD-related endpoints. These attempts were based on accumulating evidence that CVD, T2D, and cancer have overlapping risk factors and are interlinked, though the relationship is complex. For example, in a cohort of 11,941 women aged 45–50 years in Australia with three years of follow-up, an unhealthy lifestyle was associated with increased odds of accumulating multimorbidity of T2D, heart disease, and stroke, and the odds of developing two or more conditions were approximately twice as high as those of developing one new condition [74]. Some studies have also indicated that cancer and CVD share common risk factors [75, 76], possibly explained by potential mechanisms that smoking, diet, and physical activity may have common biological pathways or networks leading to the development of CVD and cancer, respectively. Although the exact underlying mechanisms remain unclear, our review provides support that lifestyle scores are highly useful to comprehensively assess the risk of multiple NCD-related outcomes simultaneously.

Although most lifestyle scores were associated with the risk of NCDs, the strength of their associations varied depending on the specific components of the lifestyle score. For HLI, we found WHT and WST seem to be better predictors than BMI for the risk of developing pancreatic cancer. This may be because central obesity is more likely to cause pancreatic cancer than overall obesity [77]. But for predicting the risk of breast cancer, BMI seems to be more powerful. A systematic review summarized that WST can predict breast cancer due to WST being closely correlated with BMI for post-menopausal women, so WST alone may not predict breast cancer [78]. Freuer [79] used a two-sample multivariable Mendelian randomization method and showed that the association between genetically predicted visceral adiposity and breast cancer was weaker than the association between general adiposity and breast cancer. In addition, we observed HLS including additional factors performed better than HLS including major factors in general. Nevertheless, incorporating the additional factors would make scores more complicated. In particular, physiological and biochemical indicators measured in a hospital could be time-consuming and costly, and would not be available for home-testing. The advantage of the simplicity of the lifestyle scores to predict disease risk would be lost. Therefore, the value of adding additional metrics requires further study and justification to balance the accuracy and accessibility of the lifestyle scores.

### Strengths and limitations

To our knowledge, this is the first systematic review summarizing the use of lifestyle scores across multiple NCD-related endpoints. The strength of the current review is that we conducted a comprehensive search, including broad search terms, multiple databases, and manual searches by score names to avoid missing available literature. However, this review has some limitations. First, the published literature focused mainly on the United States and Europe, with insufficient studies from other regions. However, because the relationship between lifestyle factors and NCDs risk can vary by ethnicity, the findings may not reflect associations across different ethnicities adequately. For example, various studies have found that for Asians, a lower BMI is associated with an increased risk of NCDs compared to the Western populations, and they recommend BMI = 24 or lower as a cut-off value for the Asian, rather than BMI = 25 used for the Western populations [80, 81]. Second, for each score, the number of studies investigating the scores and their relation to the risk of multiple NCDs was limited. Third, we were unable to provide an unbiased head-to-head comparison of these results, as they were obtained in different study populations with diverse disease endpoints. Due to the same reason, no meta-analysis could be performed, preventing us from giving a pooled estimate of relative effectiveness. Fourth, the discrepancies in lifestyle components and calculation systems of lifestyle scores observed in the primary studies may hamper the comparability of effect sizes and potentially introduce bias (e.g. diet can be assessed with validated FFQs, diet diaries, or 24-hour recalls). Therefore, it is important to note that further research is needed to fully understand the applicability of lifestyle scores and results to different ethnicities and to determine the most effective lifestyle score in a head-to-head comparison study.

## Conclusion

This systematic review provides an overview of the status and the potential of adopting lifestyle scores in the risk assessment of multiple NCDs endpoints. All 15 included lifestyle scores were shown to be useful to predict several, but not all investigated endpoints. Therefore, further research is required to determine which lifestyle score is most effective in assessing the risk of multiple NCD-related endpoints in a head-to-head study.

## Electronic supplementary material

Below is the link to the electronic supplementary material.

Supplementary Material 1: **Table S1**. PRISMA Checklist. **Material S1**. Search strategy. **Table S2**. Risk of bias assessment for the included studies. **Table S3**. Characteristics of included studies. **Table S4**. Detailed components of HLS including major factors. **Table S5**. Detailed components of UHLS including major factors. **Table S6**. Detailed components of HLS including additional factors

## Data Availability

All data generated or analysed during this study are included in this published article.

## Abbreviations

NCDs: Non-communicable diseases
DALYs: Disability-adjusted life-years
T2D: Type 2 diabetes
CVD: Cardiovascular disease
LS7: Life’s simple 7
LE8: Life’s essential 8
NOS: Newcastle–Ottawa Scale
HR: Hazard ratio
OR: Odds ratio
RR: Relative risk
CRC: Colorectal cancer
ACS: The American Cancer Society guidelines score
HLI_BMI_: Healthy lifestyle index with BMI
WCRF/AICR score: The World Cancer Research Fund/American Institute for Cancer Research score
ELIH: Empirical lifestyle pattern score for hyperinsulinemia
ELIR: Empirical lifestyle pattern score for insulin resistance
CDRI: Chronic disease risk index
LIS: Lifestyle inflammation score
ICVHMs: Ideal cardiovascular health metrics
MEDLIFE: The Mediterranean lifestyle
HLI_WST_: Healthy lifestyle index with waist circumference
HLI_WHR_: Healthy lifestyle index with a waist-to-hip ratio
HLS: Healthy lifestyle score
UHLS: Unhealthy lifestyle score
