# Supplementary material for: Lifestyle scores and their potential to estimate the risk of multiple non-communicable disease-related endpoints: a systematic review
Source: BMC Public Health. 2025 Jan 23;25:293. doi: 10.1186/s12889-025-21537-6 (PMC11758753; doi:10.1186/s12889-025-21537-6)
Supplement: Supplementary file 1 — Supplementary Material 1: Table S1. PRISMA Checklist. Material S1. Search strategy. Table S2. Risk of bias assessment for the included studies. Table S3. Characteristics of included studies. Table S4. Detailed components of HLS including major factors. Table S5. Detailed components of UHLS including major factors. Table S6. Detailed components of HLS including additional factors [file 12889_2025_21537_MOESM1_ESM.docx]

**Table S1. PRISMA Checklist [1]**

| **Section and Topic** | **Item #** | **Checklist item** | **Location where item is reported** |
| --- | --- | --- | --- |
| **TITLE** | | |  |
| Title | 1 | Identify the report as a systematic review. | Title page |
| **ABSTRACT** | | |  |
| Abstract | 2 | See the PRISMA 2020 for Abstracts checklist. | Abstract section |
| **INTRODUCTION** | | |  |
| Rationale | 3 | Describe the rationale for the review in the context of existing knowledge. | Introduction, paragraphs 1 and 2 |
| Objectives | 4 | Provide an explicit statement of the objective(s) or question(s) the review addresses. | Introduction, paragraphs 3, line 5 |
| **METHODS** | | |  |
| Eligibility criteria | 5 | Specify the inclusion and exclusion criteria for the review and how studies were grouped for the syntheses. | Methods, Inclusion and exclusion criteria |
| Information sources | 6 | Specify all databases, registers, websites, organisations, reference lists and other sources searched or consulted to identify studies. Specify the date when each source was last searched or consulted. | Methods, Search strategy, lines 2-3, 8-10 |
| Search strategy | 7 | Present the full search strategies for all databases, registers and websites, including any filters and limits used. | Material S1. Search strategy |
| Selection process | 8 | Specify the methods used to decide whether a study met the inclusion criteria of the review, including how many reviewers screened each record and each report retrieved, whether they worked independently, and if applicable, details of automation tools used in the process. | Methods, Study selection |
| Data collection process | 9 | Specify the methods used to collect data from reports, including how many reviewers collected data from each report, whether they worked independently, any processes for obtaining or confirming data from study investigators, and if applicable, details of automation tools used in the process. | Methods, Data Extraction and presentation, paragraph 1, line 1-2 |
| Data items | 10a | List and define all outcomes for which data were sought. Specify whether all results that were compatible with each outcome domain in each study were sought (e.g., for all measures, time points, analyses), and if not, the methods used to decide which results to collect. | Methods, Data Extraction and presentation, paragraph 1, lines 2-6 |
|  | 10b | List and define all other variables for which data were sought (e.g. participant and intervention characteristics, funding sources). Describe any assumptions made about any missing or unclear information. | N/A |
| Study risk of bias assessment | 11 | Specify the methods used to assess risk of bias in the included studies, including details of the tool(s) used, how many reviewers assessed each study and whether they worked independently, and if applicable, details of automation tools used in the process. | Methods, Risk of bias assessment |
| Effect measures | 12 | Specify for each outcome the effect measure(s) (e.g. risk ratio, mean difference) used in the synthesis or presentation of results. | Methods, Data extraction and presentation, paragraph 1, lines 1-5 |
| Synthesis methods | 13a | Describe the processes used to decide which studies were eligible for each synthesis (e.g. tabulating the study intervention characteristics and comparing against the planned groups for each synthesis (item #5)). | N/A |
|  | 13b | Describe any methods required to prepare the data for presentation or synthesis, such as handling of missing summary statistics, or data conversions. | N/A |
|  | 13c | Describe any methods used to tabulate or visually display results of individual studies and syntheses. | Methods, Data extraction and presentation, paragraph 1, lines 1-5 |
|  | 13d | Describe any methods used to synthesize results and provide a rationale for the choice(s). If meta-analysis was performed, describe the model(s), method(s) to identify the presence and extent of statistical heterogeneity, and software package(s) used. | N/A |
|  | 13e | Describe any methods used to explore possible causes of heterogeneity among study results (e.g. subgroup analysis, meta-regression). | N/A |
|  | 13f | Describe any sensitivity analyses conducted to assess robustness of the synthesized results. | N/A |
| Reporting bias assessment | 14 | Describe any methods used to assess risk of bias due to missing results in a synthesis (arising from reporting biases). | N/A |
| Certainty assessment | 15 | Describe any methods used to assess certainty (or confidence) in the body of evidence for an outcome. | N/A |
| **RESULTS** | | |  |
| Study selection | 16a | Describe the results of the search and selection process, from the number of records identified in the search to the number of studies included in the review, ideally using a flow diagram. | Results, Fig 1 |
|  | 16b | Cite studies that might appear to meet the inclusion criteria, but which were excluded, and explain why they were excluded. | Results, Fig 1 |
| Study characteristics | 17 | Cite each included study and present its characteristics. | Results, Components of lifestyle scores, paragraph1, lines 5-15; Characteristics of the included studies; Table S3; |
| Risk of bias in studies | 18 | Present assessments of risk of bias for each included study. | Results, Risk of bias; Table S2 |
| Results of individual studies | 19 | For all outcomes, present, for each study: (a) summary statistics for each group (where appropriate) and (b) an effect estimate and its precision (e.g. confidence/credible interval), ideally using structured tables or plots. | N/A |
| Results of syntheses | 20a | For each synthesis, briefly summarise the characteristics and risk of bias among contributing studies. | N/A |
|  | 20b | Present results of all statistical syntheses conducted. If meta-analysis was done, present for each the summary estimate and its precision (e.g. confidence/credible interval) and measures of statistical heterogeneity. If comparing groups, describe the direction of the effect. | Results, Associations between lifestyle scores and NCDs, Fig 2-4 |
|  | 20c | Present results of all investigations of possible causes of heterogeneity among study results. | N/A |
|  | 20d | Present results of all sensitivity analyses conducted to assess the robustness of the synthesized results. | N/A |
| Reporting biases | 21 | Present assessments of risk of bias due to missing results (arising from reporting biases) for each synthesis assessed. | N/A |
| Certainty of evidence | 22 | Present assessments of certainty (or confidence) in the body of evidence for each outcome assessed. | N/A |
| **DISCUSSION** | | |  |
| Discussion | 23a | Provide a general interpretation of the results in the context of other evidence. | Discussion, Principle findings and possible interpretations |
|  | 23b | Discuss any limitations of the evidence included in the review. | Discussion, Strengths and limitations, lines 5-13, 16-19 |
|  | 23c | Discuss any limitations of the review processes used. | Discussion, Strengths and limitations, line 13-16 |
|  | 23d | Discuss implications of the results for practice, policy, and future research. | Discussion, lines 6-9; Strengths and limitations, lines 19-22 |
| **OTHER INFORMATION** | | |  |
| Registration and protocol | 24a | Provide registration information for the review, including register name and registration number, or state that the review was not registered. | Material and methods, lines 2-3 |
|  | 24b | Indicate where the review protocol can be accessed, or state that a protocol was not prepared. | Material and methods, lines 2-3 |
|  | 24c | Describe and explain any amendments to information provided at registration or in the protocol. | N/A |
| Support | 25 | Describe sources of financial or non-financial support for the review, and the role of the funders or sponsors in the review. | Funding |
| Competing interests | 26 | Declare any competing interests of review authors. | Ethics declarations, Competing interests |
| Availability of data, code and other materials | 27 | Report which of the following are publicly available and where they can be found: template data collection forms; data extracted from included studies; data used for all analyses; analytic code; any other materials used in the review. | Availability of data and material |

N/A, Not applicable.

**Material S1. Search strategy**

To identify relevant studies, we searched 4 databases (PubMed, Web of Science, the Cochrane Library, Embase) using a combination of MESH terms and free text words. The MESH terms related to non-communicable disease (NCD) endpoints included "neoplasms," "diabetes mellitus, type 2," "stroke," "hypertension," "cardiovascular diseases," and "mortality" for all databases. In addition, we used "heart infarction" for Embase and "Myocardial Infarction" for the other databases. We combined these terms using the Boolean operator "OR". Next, we searched for additional terms related to study design and analysis. Specifically, we searched for MESH terms and free text words related to "lifestyle". Then we searched the words "cohort study," "case-control study," "hazard ratio," "odds ratio," "relative risk," “HR,” “OR,” and “RR.” We combined these terms separately using the "OR" operator. Finally, we combined the results of the NCD-related endpoints with the study design and analysis terms using the "AND" operator.

**PubMed**

(((((("Case-Control Stud*" OR "Case Control Stud*" OR "Case Comparison Stud*" OR "Case-Comparison Stud*" OR "Case-Compeer Stud*" OR "Case Referrent Stud*" OR "Case-Referrent Stud*" OR "Case Referent Stud*" OR "Case-Referent Stud*" OR "Case-Base Stud*" OR "Case Base Stud*" OR "Nested Case-Control Stud*" OR "Nested Case Control Stud*" OR "Matched Case-Control Stud*" OR "Matched Case Control Stud*")) OR (("Concurrent Stud*" OR "Cohort Stud*" OR "Incidence Stud*" OR "Historical Cohort Stud*" OR "Cohort Analys*" OR "Closed Cohort Stud*" OR "Birth Cohort Stud*"))) OR ("Case-Control Studies"[Mesh])) OR ("Cohort Studies"[Mesh])) AND ((((((((("Neoplasms"[Mesh] OR "Tumor*" [Title/Abstract] OR "Neoplas*" [Title/Abstract] OR "Cancer*" [Title/Abstract] OR "Malignant Neoplasm*" [Title/Abstract] OR "Malignanc*" [Title/Abstract] OR "Benign Neoplasm*" [Title/Abstract] OR "Tumour*" [Title/Abstract] OR "germ cell and embryonal neoplasms" [Title/Abstract] OR "glandular and epithelial neoplasms" [Title/Abstract] OR "hormone-dependent neoplasms" [Title/Abstract] OR "neoplasms by histologic type" [Title/Abstract] OR "neoplastic disease" [Title/Abstract] OR "neoplastic entity" [Title/Abstract] OR "neoplastic mass" [Title/Abstract] OR "tumoral entity" [Title/Abstract] OR "tumoral mass" [Title/Abstract] OR "tumorous entity" [Title/Abstract] OR "tumorous mass" [Title/Abstract] OR "tumoural mass" [Title/Abstract] OR "tumourous mass" [Title/Abstract] OR "malignant neoplasia" [Title/Abstract] OR "malignant neoplastic disease" [Title/Abstract] OR "malignant tumor" [Title/Abstract] OR "malignant tumour" [Title/Abstract] OR "acral tumor"[Title/Abstract] OR "acral tumour" [Title/Abstract] OR "embryonal and mixed neoplasms" [Title/Abstract] OR "germ cell and embryonal neoplasms"[Title/Abstract] OR "glandular and epithelial neoplasms"[Title/Abstract] OR "post-traumatic neoplasms"[Title/Abstract] OR "tumourous entity"[Title/Abstract])) OR (("Diabetes Mellitus, Type 2"[Mesh] OR "Ketosis-Resistant Diabetes Mellitus" [Title/Abstract] OR "ketosis resistant diabetes mellitus" [Title/Abstract] OR "Non-Insulin-Dependent Diabetes Mellitus" [Title/Abstract] OR "non insulin dependent diabetes" [Title/Abstract] OR "noninsulin dependent diabetes" [Title/Abstract] OR "Stable Diabetes Mellitus" [Title/Abstract] OR "NIDDM" [Title/Abstract] OR "Maturity-Onset Diabetes Mellitus" [Title/Abstract] OR "Maturity Onset Diabetes Mellitus" [Title/Abstract] OR "maturity onset diabetes" [Title/Abstract] OR "maturity onset diabetes of the young" [Title/Abstract] OR "MODY" [Title/Abstract] OR "Type 2 Diabetes Mellitus" [Title/Abstract] OR "Noninsulin-Dependent Diabetes Mellitus" [Title/Abstract] OR "Noninsulin Dependent Diabetes Mellitus" [Title/Abstract] OR "insulin independent diabetes" [Title/Abstract] OR "insulin independent diabetes mellitus" [Title/Abstract] OR "Maturity-Onset Diabetes" [Title/Abstract] OR "Maturity Onset Diabetes" [Title/Abstract] OR "Type 2 Diabetes" [Title/Abstract] OR "Adult-Onset Diabetes Mellitus" [Title/Abstract] OR "diabetes type II" [Title/Abstract] OR "dm 2" [Title/Abstract] OR "T2DM" [Title/Abstract] OR "type II diabetes" [Title/Abstract] OR "type II diabetes mellitus" [Title/Abstract] OR "adult onset diabetes" [Title/Abstract] OR "diabetes mellitus type ii" [Title/Abstract] OR "Slow-Onset Diabetes Mellitus" [Title/Abstract]))) OR (("Stroke"[Mesh] OR "Stroke*" [Title/Abstract] OR "Cerebrovascular Accident*" [Title/Abstract] OR "CVA*" [Title/Abstract] OR "Cerebrovascular Apoplexy" [Title/Abstract] OR "Brain Vascular Accident*" [Title/Abstract] OR "Cerebrovascular Stroke*" [Title/Abstract] OR "Apoplex*" [Title/Abstract] OR "Cerebral Stroke*" [Title/Abstract] OR "Acute Stroke*" [Title/Abstract] OR "Acute Cerebrovascular Accident*" [Title/Abstract] OR "acute cerebrovascular lesion" [Title/Abstract] OR "acute focal cerebral vasculopathy" [Title/Abstract] OR "apoplectic stroke" [Title/Abstract] OR "brain attack" [Title/Abstract] OR "brain insult*" [Title/Abstract] OR "cerebral insult" [Title/Abstract] OR "cerebral vascular accident" [Title/Abstract] OR "cerebral vascular insufficiency" [Title/Abstract] OR "cerebro vascular accident" [Title/Abstract] OR "cerebrovascular failure" [Title/Abstract] OR "cerebrovascular injury" [Title/Abstract] OR "cerebrovascular insufficiency" [Title/Abstract] OR "cerebrovascular insult" [Title/Abstract] OR "cryptogenic stroke" [Title/Abstract] OR "CVA" [Title/Abstract] OR "ischemic seizure" [Title/Abstract] OR "thrombotic stroke" [Title/Abstract] OR "brain accident" [Title/Abstract] OR "brain blood flow disturbance" [Title/Abstract] OR "cerebral apoplexia"[Title/Abstract] OR "cerebrovascular arrest"[Title/Abstract] OR "cerebrum vascular accident"[Title/Abstract] OR "cryptogenic stroke" [Title/Abstract]))) OR (("Myocardial Infarction"[Mesh] OR "Myocardial Infarct*" [Title/Abstract] OR "Cardiovascular Stroke*" [Title/Abstract] OR "Myocardial Infarct*" [Title/Abstract] OR "Heart Attack*" [Title/Abstract] OR "heart infarct" [Title/Abstract] OR "myocardium infarct*" [Title/Abstract] OR "second heart attack" [Title/Abstract] OR "subendocardial infarction" [Title/Abstract] OR "transmural cardiac infarction" [Title/Abstract] OR "Cardia* infarct*" [Title/Abstract] OR "heart micro infarction" [Title/Abstract] OR "heart muscle infarction" [Title/Abstract] OR "premonitory infarction sign" [Title/Abstract] OR "transmural heart infarction"[Title/Abstract]))) OR (("Hypertension"[Mesh] OR "High Blood Pressure*" [Title/Abstract] OR "acute hypertension" [Title/Abstract] OR "arterial hypertension" [Title/Abstract] OR "cardiovascular hypertension" [Title/Abstract] OR "controlled hypertension" [Title/Abstract] OR "endocrine hypertension" [Title/Abstract] OR "high renin hypertension" [Title/Abstract] OR "HTN" [Title/Abstract] OR "hypertensive disease" [Title/Abstract] OR "hypertensive effect" [Title/Abstract] OR "hypertensive response" [Title/Abstract] OR "neurogenic hypertension" [Title/Abstract] OR "preexistent hypertension" [Title/Abstract] OR "refractory hypertension" [Title/Abstract] OR "salt hypertension" [Title/Abstract] OR "secondary hypertension" [Title/Abstract] OR "systemic hypertension" [Title/Abstract] OR "salt high blood pressure" [Title/Abstract]))) OR (("Cardiovascular Diseases"[Mesh] OR cardiovascular disease* [Title/Abstract] OR angiocardiopathy [Title/Abstract] OR cardiovascular complication [Title/Abstract] OR cardiovascular diseases [Title/Abstract] OR cardiovascular disorder [Title/Abstract] OR cardiovascular disturbance [Title/Abstract] OR cardiovascular lesion [Title/Abstract] OR cardiovascular syndrome [Title/Abstract] OR cardiovascular vegetative disorder [Title/Abstract] OR major adverse cardiovascular event [Title/Abstract] OR "angiocardiovascular disease" [Title/Abstract]))) OR (("mortality"[Mesh] OR "Mortalities"[Title/Abstract] OR "Case Fatality Rate*"[Title/Abstract] OR "CFR Case Fatality Rate"[Title/Abstract] OR "Crude Death Rate*"[Title/Abstract] OR "Crude Mortality Rate*"[Title/Abstract] OR "Death Rate*"[Title/Abstract] OR "Mortality Rate*"[Title/Abstract] OR "Excess Mortalit*"[Title/Abstract] OR "Mortality Decline*"[Title/Abstract] OR "Mortality Determinant*"[Title/Abstract] OR "Differential Mortalit*"[Title/Abstract] OR "Age-Specific Death Rate*"[Title/Abstract]))) AND (((("Life Style*" [Title/Abstract] OR "Lifestyle*" [Title/Abstract] OR "Lifestyle Factor*" [Title/Abstract])) OR ("Life Style"[Mesh])) AND ("score*" [Title/Abstract] OR index* [Title/Abstract])))) AND (("hazard* ratio*"[Title/Abstract] OR "odds ratio*"[Title/Abstract] OR "relative risk*"[Title/Abstract] OR "HRs"[Title/Abstract] OR "HR"[Title/Abstract] OR "ORs"[Title/Abstract] OR "OR"[Title/Abstract] OR "RRs"[Title/Abstract] OR "RR"[Title/Abstract]))

**Web of Science**

1: TS= ((“life style*” OR “lifestyle*” OR “lifestyle factor*”) AND (“score*” OR “index*”))

2: TS= (“Tumor*” OR “Neoplas*” OR “Cancer*” OR “Malignant Neoplasm*” OR “Malignanc*” OR “Benign Neoplasm*” OR “Tumour*” OR “germ cell and embryonal neoplasms” OR “glandular and epithelial neoplasms” OR “hormone-dependent neoplasms” OR “neoplasms by histologic type” OR “neoplastic disease” OR “neoplastic entity” OR “neoplastic mass” OR “tumoral entity” OR “tumoral mass” OR “tumorous entity” OR “tumorous mass” OR “tumoural mass” OR “tumourous mass" OR “malignant neoplasia” OR “malignant neoplastic disease” OR “malignant tumor” OR “malignant tumour” OR “acral tumor” OR “acral tumour” OR “embryonal and mixed neoplasms” OR “germ cell and embryonal neoplasms” OR “glandular and epithelial neoplasms” OR “post-traumatic neoplasms” OR “tumourous entity”)

3: TS=(“Ketosis-Resistant Diabetes Mellitus” OR “ketosis resistant diabetes mellitus” OR “Non-Insulin-Dependent Diabetes Mellitus” OR “non insulin dependent diabetes” OR “noninsulin dependent diabetes” OR “Stable Diabetes Mellitus” OR “NIDDM” OR “Maturity-Onset Diabetes Mellitus” OR “Maturity Onset Diabetes Mellitus” OR “maturity onset diabetes” OR “maturity onset diabetes of the young” OR “MODY” OR “Type 2 Diabetes Mellitus” OR “Noninsulin-Dependent Diabetes Mellitus” OR “Noninsulin Dependent Diabetes Mellitus” OR “insulin independent diabetes” OR “insulin independent diabetes mellitus” OR “Maturity-Onset Diabetes” OR “Maturity Onset Diabetes” OR “Type 2 Diabetes” OR “Adult-Onset Diabetes Mellitus” OR “diabetes type II” OR “dm 2” OR “T2DM” OR “type II diabetes” OR “type II diabetes mellitus” OR “adult onset diabetes” OR “diabetes mellitus type ii” OR “Slow-Onset Diabetes Mellitus”)

4: TS=(“Stroke*” OR “Cerebrovascular Accident*” OR “CVA*” OR “Cerebrovascular Apoplexy” OR “Brain Vascular Accident*” OR “Cerebrovascular Stroke*” OR “Apoplex*” OR “Cerebral Stroke*” OR “Acute Stroke*” OR “Acute Cerebrovascular Accident*” OR “acute cerebrovascular lesion” OR “acute focal cerebral vasculopathy” OR “apoplectic stroke” OR “brain attack” OR “brain insult*” OR “cerebral insult” OR “cerebral vascular accident” OR “cerebral vascular insufficiency” OR “cerebro vascular accident” OR “cerebrovascular failure” OR “cerebrovascular injury” OR “cerebrovascular insufficiency” OR “cerebrovascular insult” OR “cryptogenic stroke” OR “CVA” OR “ischemic seizure” OR “thrombotic stroke” OR “brain accident” OR “brain blood flow disturbance” OR “cerebral apoplexia” OR “cerebrovascular arrest” OR “cerebrum vascular accident” OR “cryptogenic stroke”)

5: TS=(“Myocardial Infarct*” OR “Cardiovascular Stroke*” OR “Myocardial Infarct*” OR “Heart Attack*” OR “heart infarct” OR “myocardium infarct*” OR “second heart attack” OR “subendocardial infarction” OR “transmural cardiac infarction” OR “Cardia* infarct*” OR “heart micro infarction” OR “heart muscle infarction” OR “premonitory infarction sign” OR “transmural heart infarction”)

6: TS=(“High Blood Pressure*” OR “acute hypertension” OR “arterial hypertension” OR “cardiovascular hypertension” OR “controlled hypertension” OR “endocrine hypertension” OR “high renin hypertension” OR “HTN” OR “hypertensive disease” OR “hypertensive effect” OR “hypertensive response” OR “neurogenic hypertension” OR “preexistent hypertension” OR “refractory hypertension” OR ”salt hypertension” OR “secondary hypertension” OR “systemic hypertension” OR “salt high blood pressure”)

7: TS=(“cardiovascular disease*” OR “angiocardiopathy” OR “cardiovascular complication” OR “cardiovascular diseases” OR “cardiovascular disorder” OR “cardiovascular disturbance” OR “cardiovascular lesion” OR “cardiovascular syndrome” OR “cardiovascular vegetative disorder” OR “major adverse cardiovascular event” OR “angiocardiovascular disease”)

8: TS=(“Mortalities” OR “Case Fatality Rate*” OR “CFR Case Fatality Rate” OR “Crude Death Rate*” OR “Crude Mortality Rate*” OR “Death Rate*” OR “Mortality Rate*” OR “Excess Mortalit*” OR “Mortality Decline*” OR “Mortality Determinant*” OR “Differential Mortalit*” OR “Age-Specific Death Rate*”)

9: ALL=(“Case-Control Stud*” OR “Case Control Stud*” OR “Case Comparison Stud*” OR “Case-Comparison Stud*” OR “Case-Compeer Stud*” OR “Case Referrent Stud*” OR “Case-Referrent Stud*” OR “Case Referent Stud*” OR “Case-Referent Stud*” OR “Case-Base Stud*” OR “Case Base Stud*” OR “Nested Case-Control Stud*” OR “Nested Case Control Stud*” OR “Matched Case-Control Stud*” OR “Matched Case Control Stud*”)

10: ALL=(“Concurrent Stud*” OR “Cohort Stud*” OR “Incidence Stud*” OR “Historical Cohort Stud*” OR “Cohort Analys*” OR “Closed Cohort Stud*” OR “Birth Cohort Stud*”)

11: #8 OR #7 OR #6 OR #5 OR #4 OR #3 OR #2

12: TS=("hazard* ratio*" OR "odds ratio*" OR "relative risk*" OR "HR" OR "HRs" OR "OR" OR "ORs" OR "RR" OR "RRs")

13: #9 OR #10

14: #1 AND #11 AND #12 AND #13

**Embase**

#29. #27 AND #28

#28. 'hazard* ratio*':ab,ti OR 'odds ratio*':ab,ti OR 'relative risk*':ab,ti OR 'hr':ab,ti OR 'hrs':ab,ti OR 'or':ab,ti OR 'ors':ab,ti OR 'rr':ab,ti OR 'rrs':ab,ti

#27. #22 AND #26

#26. #23 OR #24 OR #25

#25. 'case-control stud*' OR 'case control stud*' OR 'case comparison stud*' OR 'case-comparison stud*' OR 'case-compeer stud*' OR 'case referrent stud*' OR 'case-referrent stud*' OR 'case referent stud*' OR 'case-referent stud*' OR 'case-base stud*' OR 'case base stud*' OR 'nested case-control stud*' OR 'nested case control stud*' OR 'matched case-control stud*' OR 'matched case control stud*' OR 'concurrent stud*' OR 'cohort stud*' OR 'incidence stud*' OR 'historical cohort stud*' OR 'cohort analys*' OR 'closed cohort stud*' OR 'birth cohort stud*'

#24. 'case control study'/exp

#23. 'cohort analysis'/exp

#22. #18 AND #21

#21. #2 OR #3 OR #4 OR #5 OR #6 OR #7 OR #8 OR #9 OR #10 OR #11 OR #12 OR #13 OR #19 OR #20

#20. 'mortality'/exp

#19. 'mortalities':ab,ti OR 'case fatality rate*':ab,ti OR 'cfr case fatality rate':ab,ti OR 'crude death rate*':ab,ti OR 'crude mortality rate*':ab,ti OR 'death rate*':ab,ti OR 'mortality rate*':ab,ti OR 'excess mortalit*':ab,ti OR 'mortality decline*':ab,ti OR 'mortality determinant*':ab,ti OR 'differential mortalit*':ab,ti OR 'age-specific death rate*':ab,ti

#18. #16 AND #17

#17. 'score*':ab,ti OR 'index*':ab,ti

#16. #14 OR #15

#15. 'lifestyle'/exp

#14. 'life style*':ab,ti OR 'lifestyle*':ab,ti OR 'lifestyle factor*':ab,ti

#13. 'non insulin dependent diabetes mellitus'/exp

#12. 'ketosis-resistant diabetes mellitus':ab,ti OR 'ketosis resistant diabetes mellitus':ab,ti OR 'non-insulin-dependent diabetes mellitus':ab,ti OR 'non insulin dependent diabetes':ab,ti OR 'noninsulin dependent diabetes':ab,ti OR 'stable diabetes mellitus':ab,ti OR 'niddm':ab,ti OR 'maturity-onset diabetes mellitus':ab,ti OR 'maturity onset diabetes mellitus':ab,ti OR 'maturity onset diabetes of the young':ab,ti OR 'mody':ab,ti OR 'slow-onset diabetes mellitus':ab,ti OR 'type 2 diabetes mellitus':ab,ti OR 'noninsulin-dependent diabetes mellitus':ab,ti OR 'noninsulin dependent diabetes mellitus':ab,ti OR 'insulin independent diabetes':ab,ti OR 'insulin independent diabetes mellitus':ab,ti OR 'maturity-onset diabetes':ab,ti OR 'maturity onset diabetes':ab,ti OR 'type 2 diabetes':ab,ti OR 'adult-onset diabetes mellitus':ab,ti OR 'diabetes type ii':ab,ti OR 'dm 2':ab,ti OR 't2dm':ab,ti OR 'type ii diabetes':ab,ti OR 'type ii diabetes mellitus':ab,ti OR 'adult onset diabetes':ab,ti OR 'diabetes mellitus type ii':ab,ti

#11. 'cerebrovascular accident'/exp

#10. 'stroke*':ab,ti OR 'cerebrovascular accident*':ab,ti OR 'cva*':ab,ti OR 'cerebrovascular apoplexy':ab,ti OR 'brain vascular accident*':ab,ti OR 'cerebrovascular stroke*':ab,ti OR 'apoplex*':ab,ti OR 'cerebral stroke*':ab,ti OR 'acute stroke*':ab,ti OR 'acute cerebrovascular accident*':ab,ti OR 'acute cerebrovascular lesion':ab,ti OR 'acute focal cerebral vasculopathy':ab,ti OR 'apoplectic stroke':ab,ti OR 'brain attack':ab,ti OR 'brain insult*':ab,ti OR 'cerebral insult':ab,ti OR 'cerebral vascular accident':ab,ti OR 'cerebral vascular insufficiency':ab,ti OR 'cerebro vascular accident':ab,ti OR 'cerebrovascular failure':ab,ti OR 'cerebrovascular injury':ab,ti OR 'cerebrovascular insufficiency':ab,ti OR 'cerebrovascular insult':ab,ti OR 'cva':ab,ti OR 'ischemic seizure':ab,ti OR 'thrombotic stroke':ab,ti OR 'brain accident':ab,ti OR 'brain blood flow disturbance':ab,ti OR 'cerebral apoplexia':ab,ti OR 'cerebrovascular arrest':ab,ti OR 'cerebrum vascular accident':ab,ti OR 'cryptogenic stroke':ab,ti

#9. 'heart infarction'/exp

#8. 'cardiovascular stroke*':ab,ti OR 'myocardial infarct*':ab,ti OR 'heart attack*':ab,ti OR 'heart infarct':ab,ti OR 'myocardium infarct*':ab,ti OR 'second heart attack':ab,ti OR 'subendocardial infarction':ab,ti OR 'transmural cardiac infarction':ab,ti OR 'cardia* infarct*':ab,ti OR 'heart micro infarction':ab,ti OR 'heart muscle infarction':ab,ti OR 'premonitory infarction sign':ab,ti OR 'transmural heart infarction':ab,ti

#7. 'hypertension'/exp

#6. 'high blood pressure*':ab,ti OR 'acute hypertension':ab,ti OR 'arterial hypertension':ab,ti OR 'cardiovascular hypertension':ab,ti OR 'controlled hypertension':ab,ti OR 'endocrine hypertension':ab,ti OR 'high renin hypertension':ab,ti OR 'htn':ab,ti OR 'hypertensive disease':ab,ti OR 'hypertensive effect':ab,ti OR 'hypertensive response':ab,ti OR 'neurogenic hypertension':ab,ti OR 'preexistent hypertension':ab,ti OR 'refractory hypertension':ab,ti OR 'salt hypertension':ab,ti OR 'secondary hypertension':ab,ti OR 'systemic hypertension':ab,ti OR 'salt high blood pressure':ab,ti

#5. 'cardiovascular disease'/exp

#4. 'cardiovascular disease*':ab,ti OR 'angiocardiopathy':ab,ti OR 'angiocardiovascular disease':ab,ti OR 'cardiovascular complication':ab,ti OR 'cardiovascular diseases':ab,ti OR 'cardiovascular disorder':ab,ti OR 'cardiovascular disturbance':ab,ti OR 'cardiovascular lesion':ab,ti OR 'cardiovascular syndrome':ab,ti OR 'cardiovascular vegetative disorder':ab,ti OR 'major adverse cardiovascular event':ab,ti

#3. 'neoplasm'/exp

#2. 'tumor*':ab,ti OR 'neoplas*':ab,ti OR 'cancer*':ab,ti OR 'malignant neoplasm*':ab,ti OR 'malignanc*':ab,ti OR 'benign neoplasm*':ab,ti OR 'tumour*':ab,ti OR 'hormone-dependent neoplasms':ab,ti OR 'neoplasms by histologic type':ab,ti OR 'neoplastic disease':ab,ti OR 'neoplastic entity':ab,ti OR 'neoplastic mass':ab,ti OR 'tumoral entity':ab,ti OR 'tumoral mass':ab,ti OR 'tumorous entity':ab,ti OR 'tumorous mass':ab,ti OR 'tumoural mass':ab,ti OR 'tumourous mass':ab,ti OR 'malignant neoplasia':ab,ti OR 'malignant neoplastic disease':ab,ti OR 'malignant tumor':ab,ti OR 'malignant tumour':ab,ti OR 'acral tumor':ab,ti OR 'acral tumour':ab,ti OR 'embryonal and mixed neoplasms':ab,ti OR 'germ cell and embryonal neoplasms':ab,ti OR 'glandular and epithelial neoplasms':ab,ti OR 'post-traumatic neoplasms':ab,ti OR 'tumourous entity':ab,ti

**Cochrane Library**

#1 MeSH descriptor: [Life Style] explode all trees

#2 (“Life Style*” OR “Lifestyle*” OR “Lifestyle factor*”):ti,ab

#3 #1 OR #2

#4 (“score*” OR “index*”):ti,ab

#5 #3 AND #4

#6 MeSH descriptor: [Neoplasms] explode all trees

#7 (“Tumor*” OR “Neoplas*” OR “Cancer*” OR “Malignant Neoplasm*” OR “Malignanc*” OR “Benign Neoplasm*” OR “Tumour*” OR “germ cell and embryonal neoplasms” OR “glandular and epithelial neoplasms” OR “hormone-dependent neoplasms” OR “neoplasms by histologic type” OR “neoplastic disease” OR “neoplastic entity” OR “neoplastic mass” OR “tumoral entity” OR “tumoral mass” OR “tumorous entity” OR “tumorous mass” OR “tumoural mass” OR “tumourous mass” OR “malignant neoplasia” OR “malignant neoplastic disease” OR “malignant tumor” OR “malignant tumour” OR “acral tumor” OR “acral tumour“ OR “embryonal and mixed neoplasms” OR “germ cell and embryonal neoplasms” OR “glandular and epithelial neoplasms” OR “post-traumatic neoplasms” OR “tumourous entity”):ti,ab

#8 MeSH descriptor: [Diabetes Mellitus, Type 2] explode all trees

#9 (“Ketosis-Resistant Diabetes Mellitus” OR “ketosis resistant diabetes mellitus” OR “Non-Insulin-Dependent Diabetes Mellitus” OR “non insulin dependent diabetes” OR “noninsulin dependent diabetes” OR “Stable Diabetes Mellitus” OR “NIDDM” OR “Maturity-Onset Diabetes Mellitus” OR “Maturity Onset Diabetes Mellitus” OR “maturity onset diabetes” OR “maturity onset diabetes of the young” OR “MODY” OR “Type 2 Diabetes Mellitus” OR “Noninsulin-Dependent Diabetes Mellitus” OR “Noninsulin Dependent Diabetes Mellitus” OR “insulin independent diabetes” OR “insulin independent diabetes mellitus” OR “Maturity-Onset Diabetes” OR “Maturity Onset Diabetes” OR “Type 2 Diabetes” OR “Adult-Onset Diabetes Mellitus” OR “diabetes type II” OR “dm 2” OR “T2DM” OR “type II diabetes” OR “type II diabetes mellitus” OR “adult onset diabetes” OR “diabetes mellitus type ii” OR “Slow-Onset Diabetes Mellitus”):ti,ab

#10 MeSH descriptor: [Stroke] explode all trees

#11 (“Stroke*” OR “Cerebrovascular Accident*” OR “CVA*” OR “Cerebrovascular Apoplexy” OR “Brain Vascular Accident*” OR “Cerebrovascular Stroke*” OR “Apoplex*” OR “Cerebral Stroke*” OR “Acute Stroke*” OR “Acute Cerebrovascular Accident*” OR “acute cerebrovascular lesion” OR “acute focal cerebral vasculopathy” OR “apoplectic stroke” OR “brain attack” OR “brain insult*” OR “cerebral insult” OR “cerebral vascular accident” OR “cerebral vascular insufficiency” OR “cerebro vascular accident” OR “cerebrovascular failure” OR “cerebrovascular injury” OR “cerebrovascular insufficiency” OR “cerebrovascular insult” OR “cryptogenic stroke” OR “CVA” OR “ischemic seizure” OR “thrombotic stroke”):ti,ab

#12 MeSH descriptor: [Myocardial Infarction] explode all trees

#13 (“Myocardial Infarct*” OR “Cardiovascular Stroke*” OR “Myocardial Infarct*” OR “Heart Attack*” OR “heart infarct” OR “myocardium infarct*” OR “second heart attack” OR “subendocardial infarction” OR “transmural cardiac infarction” OR “brain accident” OR “brain blood flow disturbance” OR “cerebral apoplexia” OR “cerebrovascular arrest” OR “cerebrum vascular accident” OR “cryptogenic stroke”):ti,ab 2209

#14 MeSH descriptor: [Hypertension] explode all trees

#15 (“High Blood Pressure*” OR “acute hypertension” OR “arterial hypertension” OR “cardiovascular hypertension” OR “controlled hypertension” OR “endocrine hypertension” OR “high renin hypertension” OR “HTN” OR “hypertensive disease” OR “hypertensive effect” OR “hypertensive response” OR “neurogenic hypertension” OR “preexistent hypertension” OR “refractory hypertension” OR “salt hypertension” OR “secondary hypertension” OR “systemic hypertension” OR “salt high blood pressure”):ti,ab

#16 cardiovascular diseases

#17 MeSH descriptor: [Cardiovascular Diseases] explode all trees

#18 (“cardiovascular disease*” OR “angiocardiopathy” OR “cardiovascular complication” OR “cardiovascular diseases” OR “cardiovascular disorder” OR “cardiovascular disturbance” OR “cardiovascular lesion” OR “cardiovascular syndrome” OR “cardiovascular vegetative disorder” OR “major adverse cardiovascular event” OR “angiocardiovascular disease”):ti,ab

#19 MeSH descriptor: [Mortality] explode all trees

#20 (“Mortalities” OR “Case Fatality Rate*” OR “CFR Case Fatality Rate” OR “Crude Death Rate*” OR “Crude Mortality Rate*” OR “Death Rate*” OR “Mortality Rate*” OR “Excess Mortalit*” OR “Mortality Decline*” OR “Mortality Determinant*” OR “Differential Mortalit*” OR “Age-Specific Death Rate*”):ti,ab

#21 #6 OR #7 OR #8 OR #9 OR #10 OR #11 OR #12 OR #13 OR #14 OR #15 OR #16 OR #17 OR #18 OR #19 OR #20 468415

#22 #5 AND #21

#23 MeSH descriptor: [Cohort Studies] explode all trees

#24 (“Concurrent Study” OR “Cohort Study” OR "cohort studies" OR “Incidence Study” OR “Cohort Analysis”)

#25 MeSH descriptor: [Case-Control Studies] explode all trees

#26 (“Case-Control Study” OR “Case Control Study” OR “Case Comparison Study” OR “Case Referent Study” OR “Case Base Study”)

#27 #25 OR #26

#28 #23 OR #24

#29 #27 OR #28

#30 #29 AND #22

#31 ("hazard* ratio*" OR "odds ratio*" OR "relative risk*" OR "HR" OR "HRs" OR "OR" OR "ORs" OR "RR" OR "RRs"):ti,ab

#32 #30 AND #31

**Table S2. Risk of bias assessment for the included studies**

| **First author, year** | **D1** | **D2** | **D3** | **D4** | **D5** | **D6** | **D7** | **Overall** |
| --- | --- | --- | --- | --- | --- | --- | --- | --- |
| Arthur, 2018^[2]^ | - | + | - | + | - | - | - | + |
| Arthur, 2018^[3]^ | - | + | - | + | - | + | - | + |
| Arthur, 2019^[4]^ | - | + | - | + | - | + | - | + |
| Bakhshimoghaddam, 2024^[5]^ | - | + | - | + | + | - | - | + |
| Byrd, 2020^[6]^ | + | + | - | + | + | - | - | + |
| Chen, 2021^[7]^ | + | + | - | + | - | - | - | + |
| Farhadnejad, 2021^[8]^ | + | + | + | X | - | - | X | X |
| Freisling, 2020^[9]^ | - | + | - | + | - | - | - | + |
| Fretts, 2014^[10]^ | + | + | - | + | + | - | X | X |
| Gao, 2021^[11]^ | + | + | - | + | + | - | - | + |
| Greenlee, 2017^[12]^ | - | + | - | + | - | + | - | + |
| Han, 2019^[13]^ | - | + | - | + | X | - | + | X |
| Hershey, 2020^[14]^ | - | + | - | + | X | - | - | X |
| Hosseinzadeh, 2024^[15]^ | - | + | - | + | + | - | - | + |
| Jun, 2024^[16]^ | + | X | + | - | + | - | - | X |
| Khaw, 2008^[17]^ | + | + | - | + | + | - | - | + |
| Kim, 2022^[18]^ | + | X | + | - | - | - | - | + |
| Kurth, 2006^[19]^ | + | + | - | + | + | + | - | + |
| Lau, 2021^[20]^ | - | + | - | + | - | + | - | + |
| Lee, 2024^[21]^ | + | + | - | + | + | - | - | + |
| Li, 2018^[22]^ | - | + | - | + | + | - | - | + |
| Li, 2021^[23]^ | + | + | - | + | - | - | - | + |
| Lohse, 2016^[24]^ | - | + | - | + | - | - | - | + |
| Maroto-Rodriguez, 2023^[25]^ | - | + | - | + | - | - | - | + |
| Maroto-Rodriguez,2024^[26]^ | - | + | - | + | - | - | - | + |
| Mata-Fernández, 2021^[27]^ | - | + | - | + | X | + | - | X |
| Mccullough, 2011^[28]^ | - | + | - | - | + | - | - | + |
| Mckenzie, 2015^[29]^ | - | + | - | + | + | - | - | + |
| Mckenzie, 2016^[30]^ | - | + | - | + | + | - | - | + |
| Meer, 2023^[31]^ | - | + | + | - | - | - | - | + |
| Meng, 1999^[32]^ | - | + | - | + | + | - | - | + |
| Mirizzi, 2021^[33]^ | - | + | - | + | - | - | - | + |
| Mokhtari, 2023^[34]^ | - | + | + | - | + | - | - | + |
| Myint, 2009^[35]^ | - | + | - | + | - | - | - | + |
| Naudin, 2020^[36]^ | + | + | - | + | - | - | - | + |
| Naudin, 2020^[37]^ | - | + | - | + | - | - | - | + |
| Ogunmoroti, 2016^[38]^ | + | + | - | + | - | + | - | + |
| Omrani, 2023^[39]^ | - | + | - | - | + | - | - | + |
| Peila, 2022^[40]^ | - | + | - | + | - | + | - | + |
| Peila, 2022^[41]^ | - | + | - | + | - | + | - | + |
| Peng, 2024^[42]^ | - | + | - | + | - | - | - | + |
| Plante, 2020^[43]^ | - | + | - | + | - | - | - | + |
| Rasmussen-Torvik, 2013^[44]^ | + | + | - | + | + | - | - | + |
| Sicahni, 2023^[45]^ | + | X | + | - | + | - | - | + |
| Sohouli, 2022^[46]^ | - | X | + | - | + | - | - | + |
| Sotos-Prieto, 2021^[47]^ | - | + | - | + | - | - | - | + |
| Teymoori, 2021^[48]^ | + | + | - | - | + | - | - | + |
| Teymoori, 2022^[49]^ | - | + | - | X | - | + | - | X |
| Troeschel, 2023^[50]^ | - | X | - | + | - | - | - | X |
| Viallon, 2024^[51]^ | + | + | - | + | + | - | - | + |
| Wang, 2018^[52]^ | - | + | - | + | X | - | - | X |
| Yang, 2012^[53]^ | + | + | - | + | + | - | - | + |
| Yang, 2021^[54]^ | - | X | + | - |  | - | - | X |
| Yu, 2024^[55]^ | - | + | - | + | + | - | - | + |
| Yue, 2021^[56]^ | - | + | - | + | XX | - | - | XX |
| Zhang, 2022^[57]^ | - | + | - | + | - | - | - | + |

**Domains**

D1: Risk of bias due to confounding

D2: Risk of bias arising from measurement of the exposure

D3: Risk of bias in selection of participants into the study (or into the analysis)

D4: Risk of bias due to post-exposure interventions

D5: Risk of bias due to missing data

D6: Risk of bias arising from measurement of the outcomes

D7: Risk of bias in selection of the reported results

| **Judgment** |  |
| --- | --- |
| - | Low risk of bias |
| + | Some concerns |
| X | High risk of bias |
| XX | Very high risk of bias |

**Table S3. Characteristics of included studies**

| First author, year | Population/Origin of data | Study design | Country | Sample size | Duration: mean (SD)/person-years/median | Age: mean±SD/range/midian | Sex (% male) | Ethnicity (%) |
| --- | --- | --- | --- | --- | --- | --- | --- | --- |
| Arthur, 2018^[2]^ | CSDLH | Cohort | Canada | BC: 410/1,936, EC: 177/2,519, OC: 100/2,735 | 11 | N/A | 0 | N/A |
| Arthur, 2018^[3]^ | WHI | Cohort | US | 131,833 | 16.9 | 50.0-79.0 | 0 | 83.5 White, 8.2 Black |
| Arthur, 2019^[4]^ | WHI | Cohort | US | 107,183 | 17.9 (9.0-19.4) | 50.0-79.0 | 0 | 79.99 White, 7.21 Black or African-American, 12.8 Others |
| Bakhshimoghaddam, 2024^[5]^ | SPCS | Cross-section | Iran | 2,732 | N/A | 48.29±8.53 | 41 | N/A |
| Byrd, 2020^[6]^ | NIH-AARP | Cohort | US | 453,465 | 13.5 | 61.4±5.4 | N/A | Mostly white |
| Chen, 2021^[7]^ | NOWAC | Cohort | Norway | 96,869 | 20 | 51.6±6.4 | 0 | N/A |
| Farhadnejad, 2021^[8]^ | TLGS | Cohort | Iran | 3,734 | 6.2 | 40.9±12.0 | 45.1 | N/A |
| Freisling, 2020^[9]^ | EPIC | Cohort | Denmark, Germany, Italy, the Netherlands, Spain, Sweden and the UK | 291,778 | 10.7(9.3-12.1) | 50.2±10.3 | 36 | N/A |
| Fretts, 2014^[10]^ | SHFS | Cohort | US | 1,639 | 5 | 38 | 37 | N/A |
| Gao, 2021^[11]^ | IWHS | Cohort | US | 34,254 | 18 | 61.4±4.2 | 0 | Mostly White |
| Greenlee, 2017^[12]^ | CHS | Cohort | US | 3,491 | 15 | 72.0 (64-98) | 62.8 | 86.7 White, 11.7 Black, 1.6 Others |
| Han, 2019^[13]^ | NHANES | Cohort | US | 41,357 | 5.0-6.0 | 47.3 | 48.3 | 45.5 White, 19.5 Black, 35 Others |
| Hershey, 2020^[14]^ | SUN | Cohort | Spain | 20,494 | 12.1 | 37.65±12.39 | 39.1 | N/A |
| Hosseinzadeh, 2024^[15]^ | YaHS | Cohort | Iran | 4,714 | 6 | 47.0±9.2 | 82.3 | N/A |
| Jun, 2024^[16]^ | The National Cancer Center Korea | Case-control | Korea | 919/1,846 | N/A | 56.6±9.7/56.1±9.1 | 67.7/67.7 | N/A |
| Khaw, 2008^[17]^ | EPIC-Norfolk | Cohort | UK | 20,244 | 11 | 58.1±9.3 | 45.4 | 99.5 White |
| Kim, 2022^[18]^ | The National Cancer Center | Case-control | South Korea | 923/1846 | N/A | 56.58±9.71/56.09±9.12 | 67.71/67.71 | N/A |
| Kurth, 2006^[19]^ | WHS | Cohort | US | 37,636 | 10 | 54.6±0.04 | 0 | 94.4 White, 2.1 Black, 3.5 Others |
| Lau, 2021^[20]^ | FHS and PREVEND | Cohort | US | 20,305 | 15 | 50±14 | 46 | N/A |
| Lee, 2024^[21]^ | KoGES | Cohort | Korea | 6,568 |  | 51.56±8.71 | 46.8 | N/A |
| Li, 2018^[22]^ | NHS and HPFS | Cohort | US | 123,219 | 33.9 for female, 27.2 for male | 49.0±8.20 | 36 | N/A |
| Li, 2021^[23]^ | IWHS | Cohort | US | 33,155 | 26.2 | 61.4±4.2 | 0 | Mostly White |
| Lohse, 2016^[24]^ | MONICA and NRP1A | Cohort | Switzerland | 16,722 | 21.7 | 46.1 | 48.8 | N/A |
| Maroto-Rodriguez, 2023^[25]^ | UK Biobank | Cohort | UK | 112,493 | 9.41 | 58.7±7.85 | 42.3 | 97.0 White, 3.0 Non-White |
| Maroto-Rodriguez,2024^[26]^ | UK Biobank | Cohort | UK | 110,799 | 9.42 | 58.6±7.9 | 43.9 | 96.8 White, 0.6 Mixed, 1.0 Asian, 0.8 Black, 0.3 Chinese, 0.6 Other |
| Mata-Fernández, 2021^[27]^ | SUN | Cohort | Spain | 18,419 | 11.5 (4.5) | 38.1±12.1 | 39.5 | N/A |
| Mccullough, 2011^[28]^ | CPS-II | Cohort | US | 111,966 | 14 | 62.7±6.3 | 45.5 | N/A |
| Mckenzie, 2015^[29]^ | EPIC | Cohort | Denmark, France, Germany, Greece, Italy, the Netherlands, Spain, Sweden and the UK | 242,918 | 10.9 | 53.2 | 0 | N/A |
| Mckenzie, 2016^[30]^ | EPIC | Cohort | Denmark, France, Germany, Greece, Italy, the Netherlands, Norway, Spain, Sweden and the UK | 391,608 | Women: 11.8 Men: 11.6 | 25.0-70.0 | 31 | N/A |
| Meer, 2023^[31]^ | NLCS | Case-cohort | Netherland | 485/3,767 | N/A | 60.9±3.9/61.3±4.2 | 66.0/49.5 | N/A |
| Meng, 1999^[32]^ | The Cancer Research Center of Hawaii | Cohort | US | 31,700 | 494,938 | N/A | 49.5 | 27 White, 37 Japanese, 36 Others |
| Mirizzi, 2021^[33]^ | MICOL and NUTRIHEP | Cohort | Italy | 4,866 | 68,817 | 51.50±15.8 | 51.7 | N/A |
| Mokhtari, 2023^[34]^ | Imam Hossain and Shohada hospitals | Case-control | Iran | 136/272 | N/A | 47.9±10.3 | 0 | N/A |
| Myint, 2009^[35]^ | EPIC-Norfolk | Cohort | UK | 20,040 | 11.5 | 58.3±9.2 | 44.7 | 99.5 White |
| Naudin, 2020^[36]^ | EPIC | Cohort | Denmark, France, Germany, Greece, Italy, the Netherlands, Spain, Sweden and the UK | 400,577 | 15 | 52 | 30 | N/A |
| Naudin, 2020^[37]^ | EPIC | Cohort | Denmark, France, Germany, Greece, Italy, the Netherlands, Norway, Spain, Sweden and the UK | 453,808 | 15 | 52 | 30 | N/A |
| Ogunmoroti, 2016^[38]^ | MESA | Cohort | US | 6,506 | 10.2 (9.7-10.7) | 62±10.2 | 47.2 | 39 White, 26.4 Black, 34.6 Others |
| Omrani, 2023^[39]^ | YaHS and TaMYZ | Cohort | Iran | 4,830 | 36.3 | 36±7.8 | 51 | N/A |
| Peila, 2022^[40]^ | WHI | Cohort | US | 136,945 | 16.2 (7.0) | 63.31±7.23 | 0 | 82.69 White, 8.84 Black, 8.47 Others |
| Peila, 2022^[41]^ | WHI | Cohort | US | 130,230 | 15.4 (7.2) | 63.25±7.22 | 0 | 82.85 White, 8.93 Black, 8.49 Others |
| Peng, 2024^[42]^ | UK Biobank | Cohort | UK | 277,002 | 12 | 50.2 | 45.9 | N/A |
| Plante, 2020^[43]^ | REGARDS | Cohort | US | 2,930 | 9.4 (8.5-9.9) | 61±8 | 41 | 23 Black, 77 White |
| Rasmussen-Torvik, 2013^[44]^ | ARIC | Cohort | US | 13,253 | 17.0-19.0 | 54.1±5.7 | 45.5 | 74.9 White |
| Sicahni, 2023^[45]^ | CRC surgical units of Iran | Case-control | Iran | 89/178 | N/A | 58.2±10.4/57.7±10.4 | 49.3/70 | N/A |
| Sohouli, 2022^[46]^ | The Hazrat Rasoul Hospital and Taleghani Hospital | Case-control | Iran | 253/267 | N/A | 48.91±10.46/47.13±10.08 | 0 | White |
| Sotos-Prieto, 2021^[47]^ | ENRICA | Cohort | Spain | 11,090 | 8.7 (0.87) | 46.5±16.2 | 46.8 | N/A |
| Teymoori, 2021^[48]^ | TLGS | Cohort | Iran | 4,624 | 5.7 | 40.8±12.7 | 45.1 | N/A |
| Teymoori, 2022^[49]^ | TLGS | Cohort | Iran | 4,980 | 9.1 | 46.4±11.0 | 44.7 | N/A |
| Troeschel, 2023^[50]^ | REGARDS | Cohort | US | 18,484 | 10.3 | 64.4±9.2 | 42.9 | 34.1 Black, 65.9 White |
| Viallon, 2024^[51]^ | EPIC | Cohort | Denmark, Germany, Italy, the Netherlands, Spain, Sweden and the UK | 256,769 | 16.3 | 51.4±9.3 | 38.6 | N/A |
| Wang, 2018^[52]^ | NHS and HPFS | Cohort | US | 120,007 | 2,582,699 | N/A | 38.2 | N/A |
| Yang, 2012^[53]^ | NHANES | Cohort | US | 13,312 | 182,352 | 43.4±0.9 | 48.7 | 76.4 White, 10.5 Black, 13.1 Others |
| Yang, 2021^[54]^ | NHS and HPFS | Cohort | US | 119,316 | 26 | 32.0-87.0 | 41.3 | Mostly white |
| Yu, 2024^[55]^ | UK Biobank | Cohort | UK | 170,726 | 10.9 | 55.6±8.1 | 47.3 | 95.2 White, 4.8 Others |
| Yue, 2021^[56]^ | NHSII cohort | Cohort | US | 94,217 | 24 | 46.7±7.9 | 0 | 92.9 White |
| Zhang, 2022^[57]^ | UK Biobank | Cohort | UK | 342,226 | 8.72 | 59.9±5.5 | 46.7 | 95.3 White, 4.7 Others |

Abbreviations: CSDLH: Canadian Study of Diet, Lifestyle, and Health; WHI, Women’s Health Initiative; SPCS, Sabzevar Persian Cohort Study; NIH-AARP, the National Institute of Health-American Association of Retired Persons; NOWAC, Norwegian Women and Cancer Study; TLGS, Tehran Lipid and Glucose Study; EPIC, European Prospective Investigation into Cancer and Nutrition; SHFS, Strong Heart Family Study; CHS, the Cardiovascular Health Study; NHANES, US National Health and Nutrition Examination Surveys; SUN, Seguimiento Universidad de Navarra; YaHS, Yazd Health Study; WHS, Women's Health Study; FHS, Framingham Heart Study; PREVEND, Prevention of Renal and Vascular End-Stage Disease; WHS, Women's Health Study; HPFS, Health Professionals Follow-up Study; IWHS, Iowa Women's Health Study; MONICA , Monitoring of trends and determinants in Cardiovascular disease; NRP1A, National Research Program 1A; CPS-II, Cancer Prevention Study II; NLCS, the Netherlands Cohort Study on Diet and Cancer; MICOL, The Multicentrica Italiana Colelitiasi; NUTRIHEP, The Nutrition Hepatology; MESA, Multi‐Ethnic Study of Atherosclerosis; REGARDS, Reasons for Geographic And Racial Differences in Stroke; ARIC, Atherosclerosis Risk in Communities; ENRICA: Nutrition and Cardiovascular Risk in Spain; NHS, Nurses’ Health Study; US, the United States; UK, the UK; BC, breast cancer; EC, endometrial cancer; OC, ovarian cancer; N/A, Not applicable.

**Table** **S4. Detailed components of HLS including major factors**

| **First author, year/ Lifestyle score** | **Smoking** | **Alcohol consumption** | **Diet** | **Physical activity** | **BMI** | **Scoring system** | **Total points** |
| --- | --- | --- | --- | --- | --- | --- | --- |
| **HLI_BMI_** | **Never, ex-smokers quit>10, ex-smokers quit≤10 (years), current≤15, current>15 (cigarettes/d)** | **<6, 6-<12, 12-<24, 24-<60, ≥60 (g/d)** | **Fruits and vegetables, grains, red and processed meat, the ratio of polyunsaturated to saturated fat, trans-fats, and glycemic load** | **MET-h/wk quintile values** | **<22, 22-23.9, 24-25.9, 26-29.9, ≥30 (kg/m^2^)** | **4,3,2,1,0** | **20** |
| Kurth, 2006^[19]^ | Never, past <20, past ≥20 (pack-years); current <15, current ≥15 (cigarettes/d) | 0, <1, 1-3, 4-10.5, ≥10.5 (drinks/wk) | + Folate, omega-3 fatty acids | Strenuous exercise: rarely or never, <1, 1, 2 to 3, ≥4 (times/wk) | <22.0, 22.0-24.9, 25.0-29.9, 30.0-34.9, ≥35.0 (kg/m^2^) | X | X |
| Mckenzie, 2015^[29]^ | X | 0, 0.1-4.9, 5.0-9.9, 10.0-19.9, > 20 (g/d) | + Folate, fatty fish | X | <22, 22-24, 24-26, 26-29, ≥29 (kg/m^2^) | X | X |
| Mckenzie, 2016^[30, 58]^ | X | X | X | X | X | X | X |
| Arthur, 2018^[2]^ | X | <4.9, 5.0–9.9, 10.0–19.9, 20.0–29.9, ≥30 (g/d) | X | X | X | X | X |
| Arthur, 2018^[3]^ | X | 0, >0.0-4.9, >4.9-9.9, >9.9-19.9, >19.9 (g/d) | X | X | 18.5-24.9, <18.5, 25.0-29.9, 30.0-34.9, ≥35.0 (kg/m^2^) | X | X |
| Arthur, 2019^[4]^ | Never, former≤15, former>15; current≤15, current>15 (pack-years) | X | X | X | <25.0, 25.0-29.9, 30.0-34.9, 35.0-39.9, ≥40.0 (kg/m^2^) | X | X |
| Naudin, 2020^[36]^ | X | X | X | X | 22-23.9, <22, 24-25.9, 26-29.9, ≥30 (kg/m^2^) | X | X |
| Freisling, 2020^[9]^ | Never, former, current smoker | X | mrMDS quintile | Inactive, moderately inactive, moderately active, active | 22-23.9, <22, 24-25.9, 26-29.9, ≥30 (kg/m^2^) | 4,2,0 for smoking; 4,3,1,0 for physical activity | X |
| Viallon, 2024^[51]^ |  |  |  |  |  |  |  |
| Naudin, 2020^[37]^ | X | <6, 6-11.9,12-23.9, 24-47.9, >48 (g/d) | mrMDS quintile | Inactive, moderately inactive, moderately active, active | 22-23.9, 24-25.9, <22, 26-29.9, >30 (kg/m^2^) | 4,3.2,1 for physical activity | X |
| Chen, 2021^[7]^ | X | 0, 0.1-4.9, 5.0-9.9, 10.0-19.9, >20 (g/d) | + Dairy  - The ratio of polyunsaturated to saturated fat, trans-fats, and glycemic load | Not active to very active | <23, 23-24.9, 25.0-<26.9, 27.0-29.9, ≥30 (kg/m^2^) | X | X |
| Peila, 2022^[40]^ | Never, former≤15, former>15, current≤15, current>15 (pack years) | X | AHEI:  + Nuts and soy protein, and multivitamin use  - Glycemic load | X | 18.5-24.99, 25-29.99, ≥ 30 (kg/m^2^) | WST: 2,1,0;  BMI: 2,1,0;  others: 4,3,2,1,0 | HLI_BMI_=20, HLI_WST_=18 |
| Peila, 2022^[41]^ | Never, ex-smokers quit>10, ex-smokers quit≤10 (years), current≤15, current>15 (pack years) | 0, >0-≤4.9, >4.9- ≤9.9, >9.9- ≤19.9, >19.9 (g/d) | AHEI:  + Nuts and soy protein, and multivitamin use  - Glycemic load | X | <18.5, 18.5-<25.0; 25.0-<30.0; 30.0-<35.0-≥35.0 (kg/m^2^) | X | X |
| Meer, 2023^[31]^ | X | 0, >0-≤4.9, >4.9- ≤14.9, >14.9- ≤29.9, >29.9 (g/d) | Diet Quintile Score | W:> 93.0, 66.0 – 92.9, 44.0 – 65.9, 27.0 – 43.9, 0 – 26.9 M: 116.0, 79.0 – 115.9, 51.0 – 78.9, 33.0 – 50.9, 0 – 32.9 (min/day) | X | X | X |
| **ACS guidelines score** | **N/A** | **Men>2, women>1;** **women=1, men>0 and ≤2 (drink/d); nondrinkers** | **0-2; 3-6; 7-9 point (Fruits and vegetables, whole grains, processed and red meats)** | **<8.75; 8.75 to <17.5; ≥17.5 MET-h/wk** | **>30 both times, or >30 at 1 time and 25-<30 at the other time; all others; 18.5-<25 (kg/m^2^) at both time** | **0,1,2** | **8** |
| Mccullough, 2011^[28]^ | X | X | X | X | X | X | X |
| Greenlee, 2017^[12]^ | Current smoker; quit≤1 year ago; never smoked or quit >1 year ago | X | X | 0; <8.75; ≥8.75 MET-h/wk | X | X | 10 |
| **Low-risk lifestyle score** | **Non-smoking** | **Men: 5-30, women: 5-15 (g/d)** | **AHEI score in the top 40%: high**  **intakes of vegetables, fruit, nuts, whole grains, polyunsaturated fatty acids, and long-chain omega-3 fatty acids and low intakes of red and processed meats, sugar sweetened beverages, trans fat, and sodium** | **>30 (min/d) of moderate or vigorous activities** | **18.5-24.9 (kg/m^2^)** | **Binary** | **5** |
| Li, 2018^[22]^ |  |  |  |  |  |  |  |
| **WCRF/AICR score** | **N/A** | **Yesterday: yes = 0, no = 1** | **Energy dense foods, vegetables and fruit, grains, processed meat, salt** | **Moderately physically active <1, 1, ≥2 (d/wk)** | **<18.5 or ≥30, 25-30, 18.5-24.9 (kg/m^2^)** | **0;0.5;1 or 0;1** | **9** |
| Lohse, 2016^[24]^ |  |  |  |  |  |  |  |
| **WCRF/AICR score** | **X** | **≥20, 10-20, ≤10 (g/d)** | **Energy dense foods, fast food intake, sugary drinks intake, fruits and Vegetables, cereals, whole grain bread and Legumes, white bread, pasta and rice, red and processed meat, cold meat, sodium** | **N/A** | **<18.5 or ≥30, 25-30, 18.5-24.9 (kg/m^2^)** | **0;0.5;1** | **11** |
| Mirizzi, 2021^[33]^ |  |  |  |  |  |  |  |

Abbreviations: HLI_BMI_, healthy lifestyle index with BMI; ACS guidelines score, the American Cancer Society guidelines score; WCRF/AICR score, World Cancer Research Fund and the American Institute for Cancer Research score; mrMDS, modified relative Mediterranean Diet Score; AHEI, the Alternative Healthy Eating Index; BMI, body mass index; MET, metabolic equivalent of task; N/A, Not applicable; X: same components as the most commonly used variables of a score; +: Variables added; -: Variables reduced.

**Table S5. Detailed components of UHLS including major factors**

| **First author, year/ Lifestyle score** | **Smoking** | **Alcohol consumption** | **Diet** | **Physical activity** | **BMI** | **Scoring system** | **Total points** |
| --- | --- | --- | --- | --- | --- | --- | --- |
| **ELIH** | **N/A** | **Liquor, wine** | **Positive associations: margarine, butter, red meat, fruit juice, cream soups; inverse associations, coffee, whole fruit, high-fat dairy products, snacks, salad dressing** | **Average MET-h/wk values** | **Weighting BMI** | **Coefficient wights** | **N/A** |
| Wang, 2018^[52]^ | X | X | X | X | X | X | X |
| Yang, 2021^[54]^ |  |  |  |  |  |  |  |
| Yue, 2021^[56]^ |  |  |  |  |  |  |  |
| Kim, 2022^[18]^ |  |  |  |  |  |  |  |
| Farhadnejad, 2021^[8]^ | X | N/A | - Positive associations: cream soups | X | X | X | X |
| Mokhtari, 2023^[34]^ |  |  |  |  |  |  |  |
| Sicahni, 2023^[45]^ | X | N/A | X | X | X | X | X |
| Omrani, 2023^[39]^ | X | X | X | X | X | X | X |
| **ELIR** | **N/A** | **Liquor, wine** | **Positive associations: refined grains, red meat, margarine, tomatoes, low-energy beverages, fruit juice, potatoes, processed meat, other vegetables, and tea; inverse associations: coffee, high-fat dairy products, green leafy vegetables** | **Average MET-h/wk values** | **Weighting BMI** | **Coefficient wights** | **N/A** |
| Farhadnejad, 2021^[8]^ | X | N/A | - Positive associations: potatoes | X | X | X | X |
| Kim, 2022^[18]^ | X | Liquor, wine, beer | + Positive associations: butter, fish and other sea food; inverse associations: dark yellow vegetables, and nuts | X | X | X | X |
| Teymoori, 2022^[49]^ | X | N/A | + Positive associations: butter | X | X | X | X |
| Mokhtari, 2023^[34]^ | X | N/A | -  Positive associations: low-energy beverages | X | X | X | X |
| Yang, 2021^[54]^ |  |  |  |  |  |  |  |
| **LIS** | **Current; former and never** | **Nondrinker; moderate; heavy** | **N/A** | **Low, vigorous activity 1 and moderate activity 1, or moderate activity 2-4; Vigorous activity≥2 or moderate activity ≥4 (times/wk)** | **<25, 25-29.99, ≥ 30 (kg/m^2^)** | **Coefficient weights** | **N/A** |
| Byrd, 2020^[6]^ | X | X | X | Exercises never or rarely, 1-2, ≥ 3 (times/wk) | X | X | X |
| Gao, 2021^[11]^ | X | X | X | X | X | X | X |
| Li, 2021^[23]^ |  |  |  |  |  |  |  |
| Teymoori, 2021^[48]^ | X | N/A | X | MET-min/wk tertile | X | X | X |
| Sohouli, 2022^[46]^ |  |  |  |  |  |  |  |
| Bakhshimoghaddam, 2024^[5]^ |  |  |  |  |  |  |  |
| Hosseinzadeh, 2024^[15]^ |  |  |  |  |  |  |  |
| Troeschel, 2023^[50]^ | X | X | X | Exercises never or rarely, 1-3, ≥ 4 (times/wk) | X | X | X |
| Jun, 2023^[16]^ | X | X | X | MET-min/wk tertile | <25, ≥25 (kg/m^2^) | X | X |
| Lee, 2024^[21]^ | X | X | X | 0, ＞0-<150 moderate or 75 vigorous activity; ≥150 moderate or 75 vigorous activity (min/ wk) | X | X | X |
| **CDRI** | **Current smoke (1, 1.1-1.5, 1.5 ppd), ex-smoke, never** | **Men: 1-7, women: 1-3 (drinks/wk); all others** | **Fat intake from animal products, fruit and vegetable consumption** | **N/A** | **Men: ≥32.6, 29.3-32.5, <19.6 or 24.9-29.2, 19.6-24.8; women: ≥36.0, 30.4-35.9, <18.5 or 23.7-30.3, 18.5-23.6 (kg/m^2^)** | **0,1,2,3,4** | **10** |
| Meng, 1999^[32]^ |  |  |  |  |  |  |  |
| **Health behaviours** | **Non-smoking** | **<14 units/wk** | **Fruit and vegetable intake ≥5 (servings/d)** | **Not inactive** | **N/A** | **Binary** | **4** |
| Myint, 2009^[35]^ |  |  |  |  |  |  |  |
| Khaw, 2008^[17]^ |  |  |  |  |  |  |  |

Abbreviations: ELIH, empirical lifestyle pattern score for hyperinsulinemia; ELIR, empirical lifestyle pattern score for insulin resistance; CDRI, chronic disease risk index; LIS, lifestyle inflammation score; MET, metabolic equivalent of task; N/A, Not applicable; X: same components as the most commonly used variables of a score; +: Variables added; -: Variables reduced.

**Table S6. Detailed components of HLS including additional factors**

| **First author, year/ Lifestyle score** | **Smoking** | **Alcohol consumption** | **Diet** | **Physical activity** | **BMI** | **Others** | **Scoring system** | **Total points** |
| --- | --- | --- | --- | --- | --- | --- | --- | --- |
| **LS7** | **Poor: current smoker;**  **intermediate: former smoker, quit ≤1 year; ideal: never or quit > 1 year** | **N/A** | **Poor: 0-1; intermediate: 2-3; ideal: 4-5 components (fruits and vegetables, fish, whole grains, sodium, and sugar-sweetened beverages)** | **Poor: 0, intermediate: 1-149 moderate or 1-74 vigorous, ideal: ≥ 150 moderate or ≥ 75 (min/wk) vigorous or combination** | **Poor: ≥30, intermediate: 25-29.99, ideal: <25 (kg/m^2^)** | **Total cholesterol: poor: ≥240; intermediate: 200–239 or treated to<200; ideal: <200 (mg/dL); blood pressure: poor: SBP≥140 or DBP ≥90; intermediate: SBP 120–139 or DBP 80–89 or treated to <120/80; ideal: <120/<80 (mmHg); Blood glucose poor: ≥126; intermediate:100-125; ideal: <100 (mg/dL)** | **ideal=2, intermediate=1, and poor=0 or ideal=1, others=0 (points)** | **7 or 14** |
| Ogunmoroti, 2016^[38]^ | X | X | X | X | X | X | X | X |
| Han, 2019^[13]^ |  |  |  |  |  |  |  |  |
| Plante, 2020^[43]^ |  |  |  |  |  |  |  |  |
| Fretts, 2014^[10]^ | X | X | X | Accumulated 10,000+, accumulated 3,500–10,000, accumulated <3,500 (steps/d) | X | X | X | X |
| Lau, 2021^[20]^ | X | X | X | X | Ideal: <23 (kg/m^2^) | X | Ideal=2, others=0 (points) | 14 |
| **ICVHMs** | **Never or quit>1 year** | **N/A** | **≥4 components: fruits and vegetables, fish, fiber-rich whole grains, sugar-sweetened beverages, sodium**  X | **≥150 of moderate intensity or ≥ 75 (min/wk) of vigorous intensity or equivalent combination** | **<25 (kg/m^2^)** | **Total cholesterol <200 mg/dL; blood pressure<120/<80 mmHg, fasting serum glucose<100 mg/dL** | **Binary** | **7** |
| Rasmussen-Torvik, 2013^[44]^ |  |  |  |  |  |  |  |  |
| Zhang, 2022^[57]^ | Never or previous smoking | X | **≥4 components: vegetables, fish, fiber-rich whole grains, processed meat, unprocessed meat, refined grains** | X | X | Total cholesterol<5;18mmol/L; blood pressure<120/<80 mmHg; fasting serum glucose<5.56mmol/L | X | X |
| **LE8** | never; former, current | N/A | a DASH-style eating pattern | ≥150; 120–149, 90; 90–119, 60–89; 30–59; 1–29; 0 min/wk | <25.0 kg/m^2^, 25.0–29.9, 30.0–34.9, 35.0–39.9, ≥40.0 (kg/m^2^) | sleep: 7-<9, 9-<10, 6-<7, 5-<6 or ≥10, 4-<5, <4; non-high density lipoprotein cholesterol<3.36, 3.37–4.11, 4.12–4.89, 4.90–5.66, ≥5.67; no history of diabetes and an HbA1c level <5.7, no diabetes and an HbA1c level of 5.7–6.4, diabetes with an HbA1c level <7.0, diabetes with an HbA1c level of 7.0–7.9, diabetes with an HbA1c level of 8.0–8.9, diabetes with an HbA1c level of 9.0–9.9, diabetes with an HbA1c level ≥10.0; systolic and diastolic blood pressures: <120/<80, 120–129/<80,130–139 or 80–89, 140–159 or 90–99, ≥160 or ≥100 | 0-100 | 100 (mean of individi factors ) |
| Yu, 2024^[55]^ |  |  |  |  |  |  |  |  |
| Peng, 2024^[42]^ |  |  |  |  |  |  |  |  |
| **HLI_WST_** | **Never, former≤15, former>15, current≤15, current>15 (pack years)** | **<6, 6-<12, 12-<24, 24-<60, ≥60 (g/d)** | **Fruits and vegetables, grains, red and processed meat, the ratio of polyunsaturated to saturated fat, trans-fats, nuts and soy protein, and multivitamin use** | **MET-h/wk quintile values** | **18.5-24.99, 25-29.99, ≥ 30 (kg/m^2^)** | **WST: < 80, 80-88, and ≥ 88 (cm)** | **WST: 2,1,0; others: 4,3,2,1,0** | **18** |
| Peila, 2022^[40]^ |  |  |  |  |  |  |  |  |
| **HLI_WST_** | **Never,**  **ex-smokers quit>10,**  **ex-smokers quit≤10 (years), current≤15, current>15 (pack years)** | **0, >0-≤4.9, >4.9- ≤9.9, >9.9- ≤19.9, >19.9 (g/d)** | **Fruits and vegetables, grains, red and processed meat, the ratio of polyunsaturated to saturated fat, trans-fats, nuts and soy protein, and multivitamin use** | **MET-h/wk quintile values** | **<18.5, 18.5-<25.0; 25.0-<30.0; 30.0-<35.0-≥35.0 (kg/m^2^)** | **WST: < 80, 80-88, and ≥ 88 (cm)** | **WST: 2,1,0; others: 4,3,2,1,0** | **18** |
| Peila, 2022^[41]^ |  |  |  |  |  |  |  |  |
| **HLI_WHR_** | **Never,**  **ex-smokers quit>10,**  **ex-smokers quit≤10 (years), current≤15, current>15 (cigarettes/d)** | **<6, 6-<12, 12-<24, 24-<60, ≥60 (g/d)** | **Fruits and vegetables, grains, red and processed meat, the ratio of polyunsaturated to saturated fat, trans-fats, and glycemic load** | **MET-h/wk quintile values** | **22-23.9, <22, 24-25.9, 26-29.9, ≥30 (kg/m^2^)** | **WHR: quintile** | **4,3,2,1,0** | **20** |
| Naudin, 2020^[36]^ |  |  |  |  |  |  |  |  |
| **MEDLIFE** | **N/A** | **Women: ≤0.5, men: ≤1 (serving/d)** | **Block 1: Mediterranean food consumption: sweets, red meat, processed meat, eggs, legumes, white meat, fish/seafood, potatoes, low-fat dairy products, nuts and olives, sofrito, fruit, vegetables, olive oil, cereals; Block 2: dietary habits: water and coffee, limit salt at meals, preference for whole grain products, snacks, limit snacking between meals, limit sugar in beverages** | **>300 min/wk** | **N/A** | **Nap, hours of sleep, watching TV, socializing with friends, collective ports** | **Binary** | **28** |
| Hershey, 2020^[14]^ | X | women: 0.1-5; men: 0.1-10 (g/d) | X | X | X | X | X | X |
| Mata-Fernández, 2021^[27]^ | X | X | X | X | X | X | X | X |
| Sotos-Prieto, 2021^[47]^ | X | X | -Block 2: water and coffee | >150 min/wk moderate or 60 min vigorous | X | +Eeating in company  -socializing with friends, | X | 27 |
| Maroto-Rodriguez, 2023^[25]^ | X | W: > 0- ≤ 1, M: > 0-≤ 2 (serv/d) | - Block 1: cereals  +Block 2: low salt consumption, healthy beverages consumption; -Block 2: water and coffee | 150 min of moderate, 75 min vigorous, or an equivalent combination | X | +Eating in company | X | 29 |
| Maroto-Rodrigue, 2024^[26]^ | X | W: > 0- ≤ 1, M: > 0-≤ 2 (serv/d) | -Block 1: cereals | 150 min of moderate, 75 min vigorous, or an equivalent combination | X | +Eating in company | X | 29 |

Abbreviations: LS7, life's simple 7; ICVHMs, ideal cardiovascular health metrics; LE8, Life’s Essential 8; MEDLIFE, the Mediterranean lifestyle; HLI, healthy lifestyle index; BMI, body mass index; WST, waist circumference; WHR, waist-to-hip ratio; MedD; mediterranean diet; MET, metabolic equivalent of task; N/A, Not applicable; X: same components as the most commonly used variables of a score; +: Variables added; -: Variables reduced.

**References**

1. Page MJ, McKenzie JE, Bossuyt PM, Boutron I, Hoffmann TC, Mulrow CD *et al*: The PRISMA 2020 statement: an updated guideline for reporting systematic reviews. *Bmj* 2021, 372:n71.

2. Arthur R, Kirsh VA, Kreiger N, Rohan TJCC, Control: A healthy lifestyle index and its association with risk of breast, endometrial, and ovarian cancer among Canadian women. 2018, 29:485-493.

3. Arthur R, Wassertheil-Smoller S, Manson JE, Luo J, Snetselaar L, Hastert T *et al*: The Combined Association of Modifiable Risk Factors with Breast Cancer Risk in the Women's Health Initiative. *Cancer prevention research (Philadelphia, Pa)* 2018, 11(6):317-326.

4. Arthur R, Brasky TM, Crane TE, Felix AS, Kaunitz AM, Shadyab AH *et al*: Associations of a healthy lifestyle index with the risks of endometrial and ovarian cancer among women in the Women’s Health Initiative Study. 2019, 188(2):261-273.

5. Bakhshimoghaddam F, Jafarirad S, Maraghi E, Ghorat F: Association of dietary and lifestyle inflammation score with type 2 diabetes mellitus and cardiometabolic risk factors in Iranian adults: Sabzevar Persian Cohort Study. *The British journal of nutrition* 2024, 131(3):521-530.

6. Byrd DA, Judd SE, Flanders WD, Hartman TJ, Fedirko V, Agurs-Collins T *et al*: Associations of novel dietary and lifestyle inflammation scores with incident colorectal cancer in the NIH-AARP Diet and Health Study. 2020, 4(3):pkaa009.

7. Chen SL, Braaten T, Borch KB, Ferrari P, Sandanger TM, Nøst THJCE: Combined lifestyle behaviors and the incidence of common cancer types in the Norwegian Women and Cancer Study (NOWAC). 2021:721-734.

8. Farhadnejad H, Mokhtari E, Teymoori F, Sohouli MH, Moslehi N, Mirmiran P *et al*: Association of the insulinemic potential of diet and lifestyle with risk of diabetes incident in Tehranian adults: a population based cohort study. 2021, 20(1):1-10.

9. Freisling H, Viallon V, Lennon H, Bagnardi V, Ricci C, Butterworth AS *et al*: Lifestyle factors and risk of multimorbidity of cancer and cardiometabolic diseases: a multinational cohort study. 2020, 18:1-11.

10. Fretts AM, Howard BV, McKnight B, Duncan GE, Beresford SA, Mete M *et al*: Life's Simple 7 and incidence of diabetes among American Indians: the Strong Heart Family Study. *Diabetes care* 2014, 37(8):2240-2245.

11. Gao Y, Byrd DA, Prizment A, Lazovich D, Bostick RM: Associations of Novel Lifestyle- and Whole Foods-Based Inflammation Scores with Incident Colorectal Cancer Among Women. *Nutrition and cancer* 2022, 74(4):1356-1369.

12. Greenlee H, Strizich G, Lovasi GS, Kaplan RC, Biggs ML, Li CI *et al*: Concordance With Prevention Guidelines and Subsequent Cancer, Cardiovascular Disease, and Mortality: A Longitudinal Study of Older Adults. *American journal of epidemiology* 2017, 186(10):1168-1179.

13. Han L, You D, Ma W, Astell-Burt T, Feng X, Duan S *et al*: National trends in American Heart Association revised life's simple 7 metrics associated with risk of mortality among US adults. 2019, 2(10):e1913131-e1913131.

14. Hershey MS, Fernandez-Montero A, Sotos-Prieto M, Kales S, Gea A, Ruiz-Estigarribia L *et al*: The Association Between the Mediterranean Lifestyle Index and All-Cause Mortality in the Seguimiento Universidad de Navarra Cohort. *American journal of preventive medicine* 2020, 59(6):e239-e248.

15. Hosseinzadeh M, Saber N, Bidar SS, Hashemi S, Teymoori F, Mirzaei M *et al*: Association of dietary and lifestyle inflammatory indices with type 2 diabetes risk in Iranian adults. *BMC Endocr Disord* 2024, 24(1):131.

16. Jun S, Lee J, Oh JH, Chang HJ, Sohn DK, Shin A *et al*: Association of the inflammatory balance of diet and lifestyle with colorectal cancer among Korean adults: a case-control study. *Annals of Nutrition and Metabolism* 2023, 79:721.

17. Khaw K-T, Wareham N, Bingham S, Welch A, Luben R, Day NJPm: Combined impact of health behaviours and mortality in men and women: the EPIC-Norfolk prospective population study. 2008, 5(1):e12.

18. Kim J, Lee J, Oh JH, Chang HJ, Sohn DK, Shin A *et al*: Interactive effect of the empirical lifestyle index for insulin resistance with the common genetic susceptibility locus rs2423279 for colorectal cancer. 2022:1-27.

19. Kurth T, Moore SC, Gaziano JM, Kase CS, Stampfer MJ, Berger K *et al*: Healthy lifestyle and the risk of stroke in women. 2006, 166(13):1403-1409.

20. Lau ES, Paniagua SM, Liu E, Jovani M, Li SX, Takvorian K *et al*: Cardiovascular risk factors are associated with future cancer. 2021, 3(1):48-58.

21. Lee HA, Park H, Park B: Genetic predisposition, lifestyle inflammation score, food-based dietary inflammatory index, and the risk for incident diabetes: Findings from the KoGES data. *Nutrition, Metabolism and Cardiovascular Diseases* 2024, 34(3):642-650.

22. Li Y, Pan A, Wang DD, Liu X, Dhana K, Franco OH *et al*: Impact of healthy lifestyle factors on life expectancies in the US population. 2018, 138(4):345-355.

23. Li Z, Gao Y, Byrd DA, Gibbs DC, Prizment AE, Lazovich D *et al*: Novel dietary and lifestyle inflammation scores directly associated with all-cause, all-cancer, and all-cardiovascular disease mortality risks among women. 2021, 151(4):930-939.

24. Lohse T, Faeh D, Bopp M, Rohrmann S, nutrition SNCSGJTAjoc: Adherence to the cancer prevention recommendations of the World Cancer Research Fund/American Institute for Cancer Research and mortality: a census-linked cohort. 2016, 104(3):678-685.

25. Maroto-Rodriguez J, Ortolá R, Carballo-Casla A, Iriarte-Campo V, Salinero-Fort M, Rodríguez-Artalejo F *et al*: Association between a mediterranean lifestyle and Type 2 diabetes incidence: a prospective UK biobank study. *Cardiovascular diabetology* 2023, 22(1):271.

26. Maroto-Rodriguez J, Delgado-Velandia M, Ortolá R, Perez-Cornago A, Kales SN, Rodríguez-Artalejo F *et al*: Association of a Mediterranean Lifestyle With All-Cause and Cause-Specific Mortality: A Prospective Study from the UK Biobank. *Mayo Clin Proc* 2024, 99(4):551-563.

27. Mata-Fernández A, Hershey MS, Pastrana-Delgado JC, Sotos-Prieto M, Ruiz-Canela M, Kales SN *et al*: A Mediterranean lifestyle reduces the risk of cardiovascular disease in the "Seguimiento Universidad de Navarra" (SUN) cohort. *Nutrition, metabolism, and cardiovascular diseases : NMCD* 2021, 31(6):1728-1737.

28. McCullough ML, Patel AV, Kushi LH, Patel R, Willett WC, Doyle C *et al*: Following cancer prevention guidelines reduces risk of cancer, cardiovascular disease, and all-cause mortality. *Cancer epidemiology, biomarkers & prevention : a publication of the American Association for Cancer Research, cosponsored by the American Society of Preventive Oncology* 2011, 20(6):1089-1097.

29. McKenzie F, Ferrari P, Freisling H, Chajès V, Rinaldi S, de Batlle J *et al*: Healthy lifestyle and risk of breast cancer among postmenopausal women in the European Prospective Investigation into Cancer and Nutrition cohort study. *International journal of cancer* 2015, 136(11):2640-2648.

30. McKenzie F, Biessy C, Ferrari P, Freisling H, Rinaldi S, Chajès V *et al*: Healthy lifestyle and risk of cancer in the European prospective investigation into cancer and nutrition cohort study. 2016, 95(16).

31. Meer R, van de Pol J, van den Brandt PA, Schouten LJ: The association of healthy lifestyle index score and the risk of renal cell cancer in the Netherlands cohort study. *BMC Cancer* 2023, 23(1):156.

32. Meng L, Maskarinec G, Lee J, Kolonel LNJPm: Lifestyle factors and chronic diseases: application of a composite risk index. 1999, 29(4):296-304.

33. Mirizzi A, Aballay LR, Misciagna G, Caruso MG, Bonfiglio C, Sorino P *et al*: Modified wcrf/aicr score and all-cause, digestive system, cardiovascular, cancer and other-cause-related mortality: A competing risk analysis of two cohort studies conducted in southern italy. 2021, 13(11):4002.

34. Mokhtari E, Jamshidi S, Daftari G, Farhadnejad H, Teymoori F, Momeni SA *et al*: The relationship between the insulinemic potential of diet and lifestyle and risk of breast cancer: a case-control study among iranian adult women. *Archives of public health = Archives belges de sante publique* 2023, 81(1):4.

35. Myint PK, Luben RN, Wareham NJ, Bingham SA, Khaw K-TJB: Combined effect of health behaviours and risk of first ever stroke in 20 040 men and women over 11 years’ follow-up in Norfolk cohort of European Prospective Investigation of Cancer (EPIC Norfolk): prospective population study. 2009, 338.

36. Naudin S, Viallon V, Hashim D, Freisling H, Jenab M, Weiderpass E *et al*: Healthy lifestyle and the risk of pancreatic cancer in the EPIC study. 2020, 35:975-986.

37. Naudin S, Solans Margalef M, Saberi Hosnijeh F, Nieters A, Kyrø C, Tjønneland A *et al*: Healthy lifestyle and the risk of lymphoma in the European prospective investigation into cancer and nutrition study. 2020, 147(6):1649-1656.

38. Ogunmoroti O, Allen NB, Cushman M, Michos ED, Rundek T, Rana JS *et al*: Association Between Life's Simple 7 and Noncardiovascular Disease: The Multi-Ethnic Study of Atherosclerosis. *Journal of the American Heart Association* 2016, 5(10).

39. Omrani M, Hosseinzadeh M, Shab Bidar S, Mirzaei M, Teymoori F, Nadjarzadeh A *et al*: Insulinaemic potential of diet and lifestyle and risk of type 2 diabetes in the Iranian adults: result from Yazd health study. *BMC Endocr Disord* 2023, 23(1):136.

40. Peila R, Coday M, Crane TE, Saquib N, Shadyab AH, Tabung FK *et al*: Healthy lifestyle index and risk of pancreatic cancer in the Women’s Health Initiative. 2022, 33(5):737-747.

41. Peila R, Lane DS, Shadyab AH, Saquib N, Strickler HD, Manson JE *et al*: Healthy lifestyle index and the risk of ductal carcinoma in situ of the breast in the Women's Health Initiative. 2022, 151(4):526-538.

42. Peng Y, Wang P, Du H, Liu F, Wang X, Si C *et al*: Cardiovascular health, polygenic risk score, and cancer risk: a prospective cohort study. *Am J Clin Nutr* 2024.

43. Plante TB, Koh I, Judd SE, Howard G, Howard VJ, Zakai NA *et al*: Life’s simple 7 and incident hypertension: the REGARDS study. 2020, 9(19):e016482.

44. Rasmussen-Torvik LJ, Shay CM, Abramson JG, Friedrich CA, Nettleton JA, Prizment AE *et al*: Ideal cardiovascular health is inversely associated with incident cancer: the Atherosclerosis Risk In Communities study. *Circulation* 2013, 127(12):1270-1275.

45. Sicahni PH, Makhtoomi M, Leilami K, Shateri Z, Mohammadi F, Nouri M *et al*: Dietary and lifestyle indices for hyperinsulinemia and colorectal cancer risk: a case-control study. *BMC Gastroenterol* 2023, 23(1):434.

46. Sohouli MH, Hadizadeh M, Mardali F, Sanati V, da Silva Magalhães EI, Zarrati MJNJ: Association between novel dietary and lifestyle inflammation indices with risk of breast cancer (BrCa): a case–control study. 2022, 21(1):14.

47. Sotos-Prieto M, Ortolá R, Ruiz-Canela M, Garcia-Esquinas E, Martínez-Gómez D, Lopez-Garcia E *et al*: Association between the Mediterranean lifestyle, metabolic syndrome and mortality: a whole-country cohort in Spain. *Cardiovascular diabetology* 2021, 20(1):5.

48. Teymoori F, Farhadnejad H, Mokhtari E, Sohouli MH, Moslehi N, Mirmiran P *et al*: Dietary and lifestyle inflammatory scores and risk of incident diabetes: a prospective cohort among participants of Tehran lipid and glucose study. 2021, 21:1-12.

49. Teymoori F, Mokhtari E, Farhadnejad H, Mirmiran P, Rad HA, Azizi FJN, Metabolism *et al*: The dietary and lifestyle indices of insulin resistance are associated with increased risk of cardiovascular diseases: A prospective study among an Iranian adult population. 2022, 32(9):2216-2226.

50. Troeschel AN, Byrd DA, Judd S, Flanders WD, Bostick RM: Associations of dietary and lifestyle inflammation scores with mortality due to CVD, cancer, and all causes among Black and White American men and women. *The British journal of nutrition* 2023, 129(3):523-534.

51. Viallon V, Freisling H, Matta K, Nannsen A, Dahm CC, Tjønneland A *et al*: On the use of the healthy lifestyle index to investigate specific disease outcomes. *Sci Rep* 2024, 14(1):16330.

52. Wang W, Fung TT, Wang M, Smith-Warner SA, Giovannucci EL, Tabung FKJJcs: Association of the insulinemic potential of diet and lifestyle with risk of digestive system cancers in men and women. 2018, 2(4):pky080.

53. Yang Q, Cogswell ME, Flanders WD, Hong Y, Zhang Z, Loustalot F *et al*: Trends in cardiovascular health metrics and associations with all-cause and CVD mortality among US adults. *Jama* 2012, 307(12):1273-1283.

54. Yang W, Sui J, Zhao L, Ma Y, Tabung FK, Simon TG *et al*: Association of Inflammatory and Insulinemic Potential of Diet and Lifestyle with Risk of Hepatocellular Carcinoma. *Cancer epidemiology, biomarkers & prevention : a publication of the American Association for Cancer Research, cosponsored by the American Society of Preventive Oncology* 2021, 30(4):789-796.

55. Yu YT, Sun Y, Yu YF, Wang YY, Chen C, Tan X *et al*: Life's Essential 8 and risk of non-communicable chronic diseases: Outcome-wide analyses. *Chinese Medical Journal* 2024, 137(13):1553-1562.

56. Yue Y, Hur J, Cao Y, Tabung F, Wang M, Wu K *et al*: Prospective evaluation of dietary and lifestyle pattern indices with risk of colorectal cancer in a cohort of younger women. 2021, 32(6):778-786.

57. Zhang J, Yu H, Huang T, Huang N, Liang H: Importance of ideal cardiovascular health metrics in the risk of colorectal cancer among people aged 50 years or older: a UK Biobank cohort study. *BMJ open* 2022, 12(5):e059642.

58. McKenzie F, Biessy C, Ferrari P, Freisling H, Rinaldi S, Chajès V *et al*: Healthy Lifestyle and Risk of Cancer in the European Prospective Investigation Into Cancer and Nutrition Cohort Study. *Medicine (Baltimore)* 2016, 95(16):e2850.
